# Characterization of nucleic acids from extracellular vesicle-enriched human sweat

**DOI:** 10.1186/s12864-021-07733-9

**Published:** 2021-06-09

**Authors:** Geneviève Bart, Daniel Fischer, Anatoliy Samoylenko, Artem Zhyvolozhnyi, Pavlo Stehantsev, Ilkka Miinalainen, Mika Kaakinen, Tuomas Nurmi, Prateek Singh, Susanna Kosamo, Lauri Rannaste, Sirja Viitala, Jussi Hiltunen, Seppo J Vainio

**Affiliations:** 1 Faculty of Biochemistry and Molecular Medicine, Disease Networks Research Unit, Laboratory of Developmental Biology, Kvantum Institute, Infotech Oulu, University of Oulu, 90014 University of Oulu, Oulu, Finland; 2 Production Systems, Natural Resources Institute Finland (LUKE), 31600 Jokioinen, Finland; 3 Present Address: Finnadvance, Aapistie 5, 90220 Oulu, Finland; 4 Biosensors, VTT, Technical Research Center of Finland Ltd, Kaitoväylä 1, 90570 Oulu, Finland

**Keywords:** Extracellular vesicles (EV), Sweat, Genomics, Transcriptomics, Exercise, Microbiome, Metagenomics, Skin

## Abstract

**Background:**

The human sweat is a mixture of secretions from three types of glands: eccrine, apocrine, and sebaceous. Eccrine glands open directly on the skin surface and produce high amounts of water-based fluid in response to heat, emotion, and physical activity, whereas the other glands produce oily fluids and waxy sebum. While most body fluids have been shown to contain nucleic acids, both as ribonucleoprotein complexes and associated with extracellular vesicles (EVs), these have not been investigated in sweat. In this study we aimed to explore and characterize the nucleic acids associated with sweat particles.

**Results:**

We used next generation sequencing (NGS) to characterize DNA and RNA in pooled and individual samples of EV-enriched sweat collected from volunteers performing rigorous exercise. In all sequenced samples, we identified DNA originating from all human chromosomes, but only the mitochondrial chromosome was highly represented with 100% coverage. Most of the DNA mapped to unannotated regions of the human genome with some regions highly represented in all samples. Approximately 5 % of the reads were found to map to other genomes: including bacteria (83%), archaea (3%), and virus (13%), identified bacteria species were consistent with those commonly colonizing the human upper body and arm skin. Small RNA-seq from EV-enriched pooled sweat RNA resulted in 74% of the trimmed reads mapped to the human genome, with 29% corresponding to unannotated regions. Over 70% of the RNA reads mapping to an annotated region were tRNA, while misc. RNA (18,5%), protein coding RNA (5%) and miRNA (1,85%) were much less represented. RNA-seq from individually processed EV-enriched sweat collection generally resulted in fewer percentage of reads mapping to the human genome (7–45%), with 50–60% of those reads mapping to unannotated region of the genome and 30–55% being tRNAs, and lower percentage of reads being rRNA, LincRNA, misc. RNA, and protein coding RNA.

**Conclusions:**

Our data demonstrates that sweat, as all other body fluids, contains a wealth of nucleic acids, including DNA and RNA of human and microbial origin, opening a possibility to investigate sweat as a source for biomarkers for specific health parameters.

**Supplementary Information:**

The online version contains supplementary material available at 10.1186/s12864-021-07733-9.

## Background

Sweat is a biofluid continuously produced by skin glands for secretion to the body surface. Unlike urine, which accumulates in the bladder over time, and is flushed out only when the bladder is emptied, sweat is released continuously, from less than 1 pL/minute in resting conditions to several nL/minute per gland during exercise [1], and could therefore be collected non-invasively for analysis. In addition to changes in the sweat release rate, the composition of sweat is altered by physical activity and presence of health conditions. Detection of specific metabolites, ions, hormones, peptides, cytokines, and glucose in sweat has potential diagnostic value. Glucose levels in sweat reflect changes in the blood glucose level, and this observation has led to development of non-invasive glucose monitoring methods [2–4]. The sweat proteome has been shown to be different between healthy subjects and people with schizophrenia [5], and between healthy people and patients with active tuberculosis [6]. The presence of viral particles in sweat has also been reported, consisting mostly in infectious viruses such as papilloma or polyoma virus and bacteriophages [7], but other infective viruses like Hepatitis C virus have also been detected [8]. Sweat analysis for forensic purposes has also been reported [9, 10], but while saliva is routinely used for genotyping, no genetic tests based on sweat nucleic acids have been published beyond finding specific markers to distinguish sweat from other biofluids [11].

In addition to ions and macromolecules, biofluids carry insoluble particles containing nucleic acids, including lipid droplets [12], ribonucleoprotein complexes, extracellular vesicles (EVs) and whole cells. Systematic studies of sweat EV cargo are difficult, because of the mixtures of environmental contaminants on the skin surface, and because most of the collection methods interfere with the normal sweating process [13]. Sweat contains several types of EVs: apoptotic bodies from holocrine secretion of sebaceous glands [14], large membrane vesicles from axillary apocrine glands [15], and 100-200 nm EVs with CD63, CD9 and CD81 tetraspanins [16, 17].

Sweat secretion is qualitatively and quantitatively affected by stimuli such as heat, exercise, emotions, and health status. We recently reported differential sweat EV miRNA secretion in relation to specific exercise [17], supporting the notion that exercise-induced sweat could be used as a source of biomarkers for sport practice.

Both cell free DNA (cfDNA) and extracellular RNA (exRNA) have shown great promise as biomarkers (Reviewed in [18]), therefore our aim was to characterize the nucleic acids associated with sweat EVs. Because our study design was exploratory, our goal was to obtain large quantities of starting material for inventory from the study subjects, and we initially pooled sweat from 13 individuals for nucleic acid analysis. We subsequently also extracted DNA and RNA from sweat of individual collection for analysis. We found human DNA fragments mapping to all chromosomes, but most of the DNA originated from unannotated regions of the human genome. Non-human DNA was found to be derived from skin microbiota, mainly bacteria, but also archaea and viruses. EV-associated RNA species contained a high proportion of tRNA, rRNA and miscRNA, and also approximately 89 miRNA and more than 500 mRNA species. In addition, our NGS data shows the presence of RNA of microbial, fungal and viral origin.

To our knowledge, this is the first published study characterizing EV-associated nucleic acids in exercise induced human sweat.

## Results

### Sweat collection and processing

**Fig. 1:**
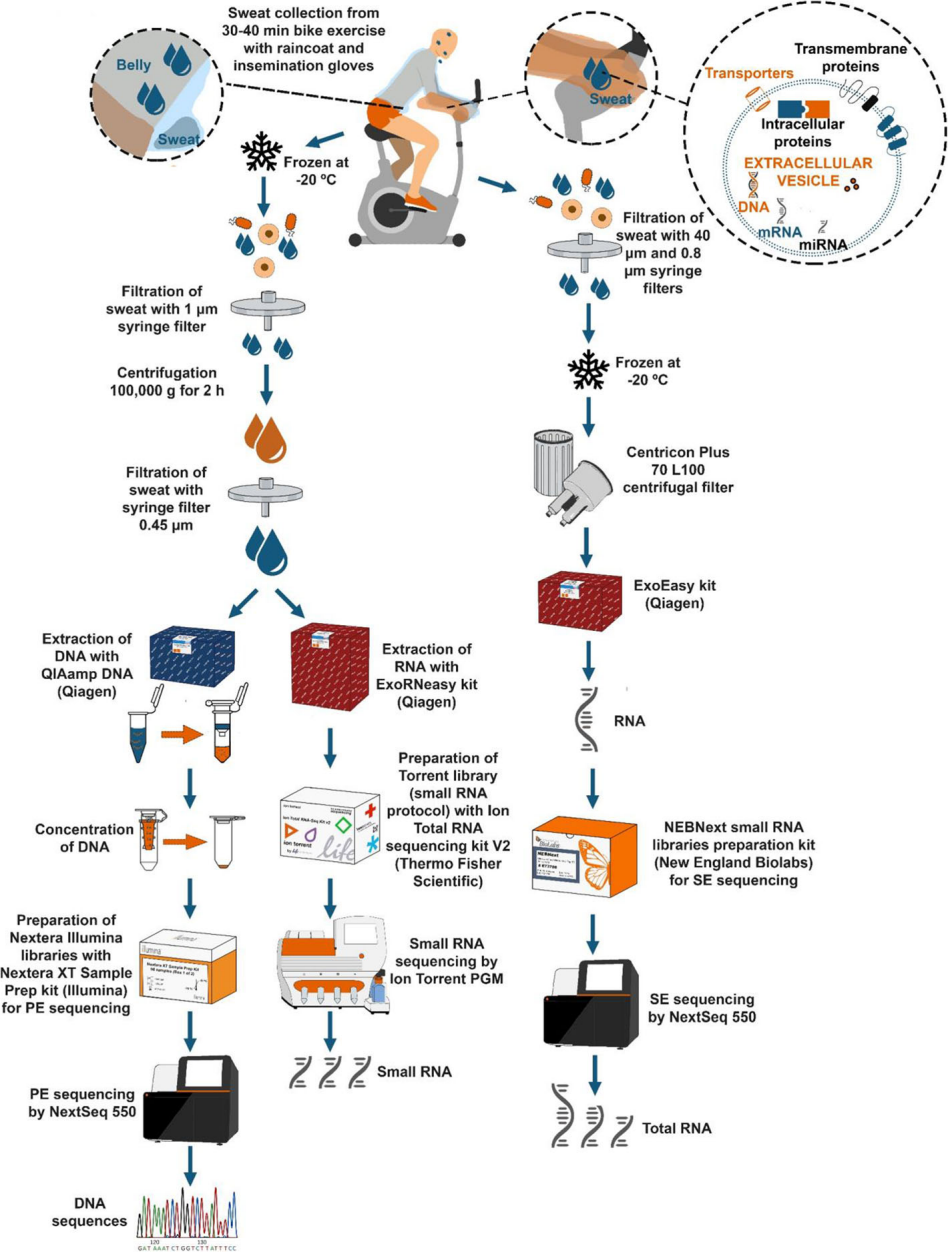
Workflow. Description of the workflow: left side preparation of EV-enriched sweat DNA. Middle preparation of EV-enriched Sweat RNA from pool for small RNA-seq. Right preparation of EV-enriched sweat RNA from individuals

We collected sweat from people undergoing vigorous biking exercise. We first collected 1,4 l of sweat from 13 volunteers of both gender aged from 26 to 56 years at the time of collection, amounts of individual collections were not recorded, the sweat was stored at − 20 °C and mixed after thawing for processing to DNA and RNA for sequencing (Fig. 1, left side). We collected sweat for RNA from 25 individuals during a 30 min biking exercise, with the amount of sweat collected from each individual ranging from 6 to 175 ml (Table 1), these collections were processed individually to NGS (Fig. 1), or EV characterization.

**Table 1:**
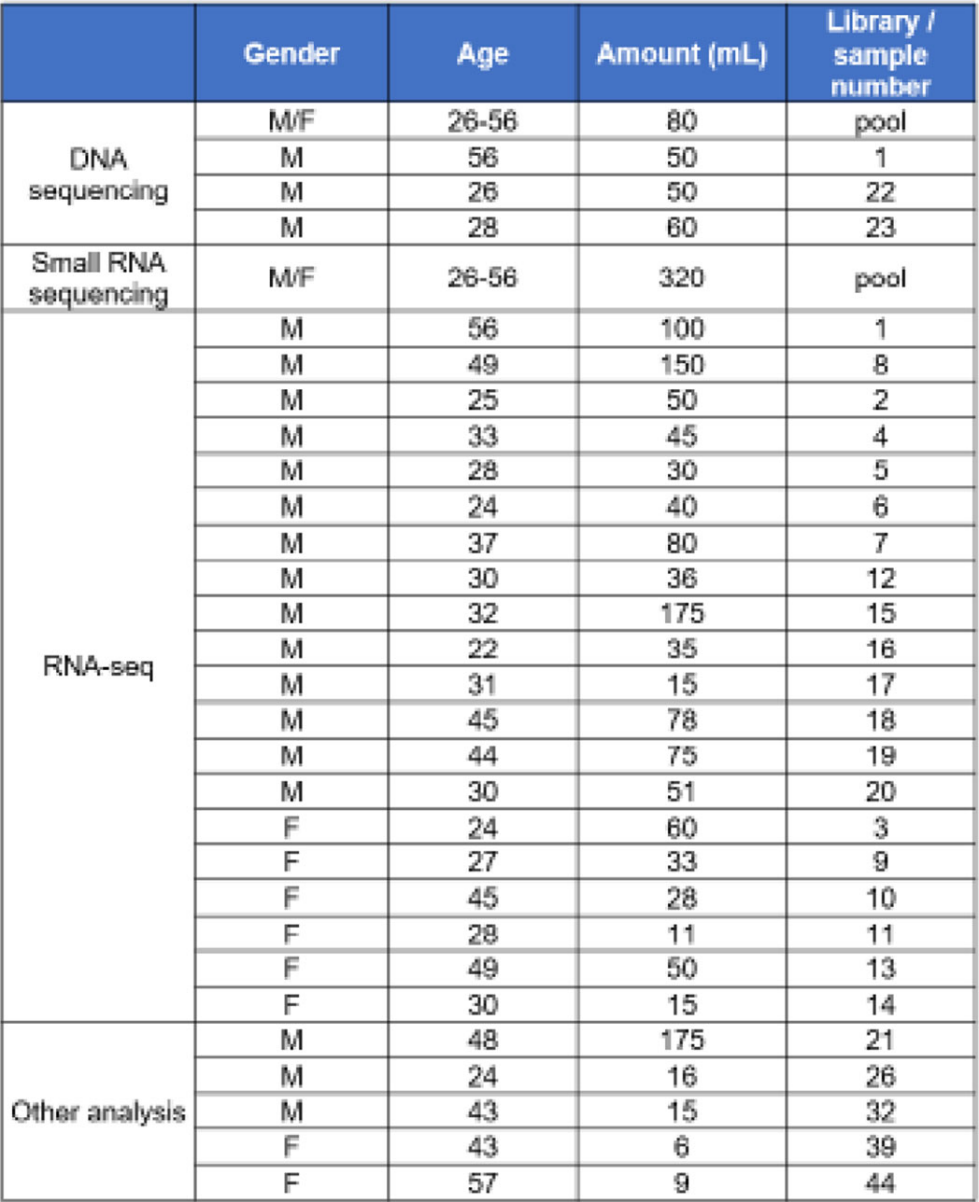
Sample Information. Gender, age, sweat volume, and library assignment for each sample

### DNA isolation and NGS library preparation

**Fig. 2:**
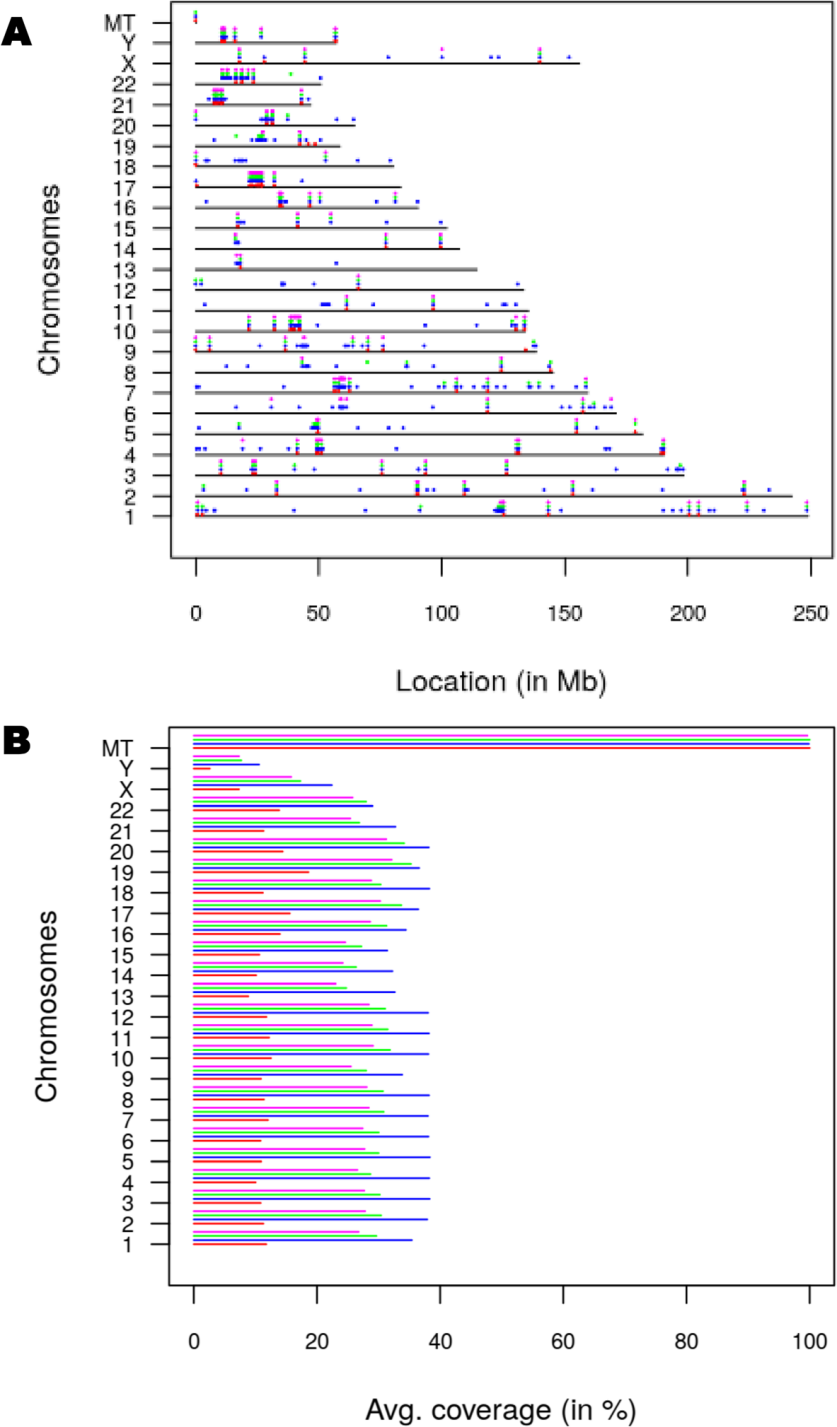
DNA sequencing results. **A**: distribution of reads on each chromosome **B**: coverage for each chromosome. Individual sample indicated by color

We used DNA from the pooled sample and from three individual collections for whole genome sequencing. The total amount of double stranded DNA recovered was small with a range of 3 to 11 ng total DNA. We chose to make pair-ended libraries with a small genome library kit from Illumina. We were able to get between 10 and 20 M reads per sample. Alignment of the reads to the human genome (GRch38) showed small coverage with some clear hot spots where high number of reads from all samples were detected (Fig. 2A). Coverage on each chromosome (10–30%) appeared to be dependent of sequencing depth, with the notable exception of the mitochondrial chromosome, which was entirely covered in all samples (Fig. 2B).

### DNA sequencing analysis

**Fig. 3:**
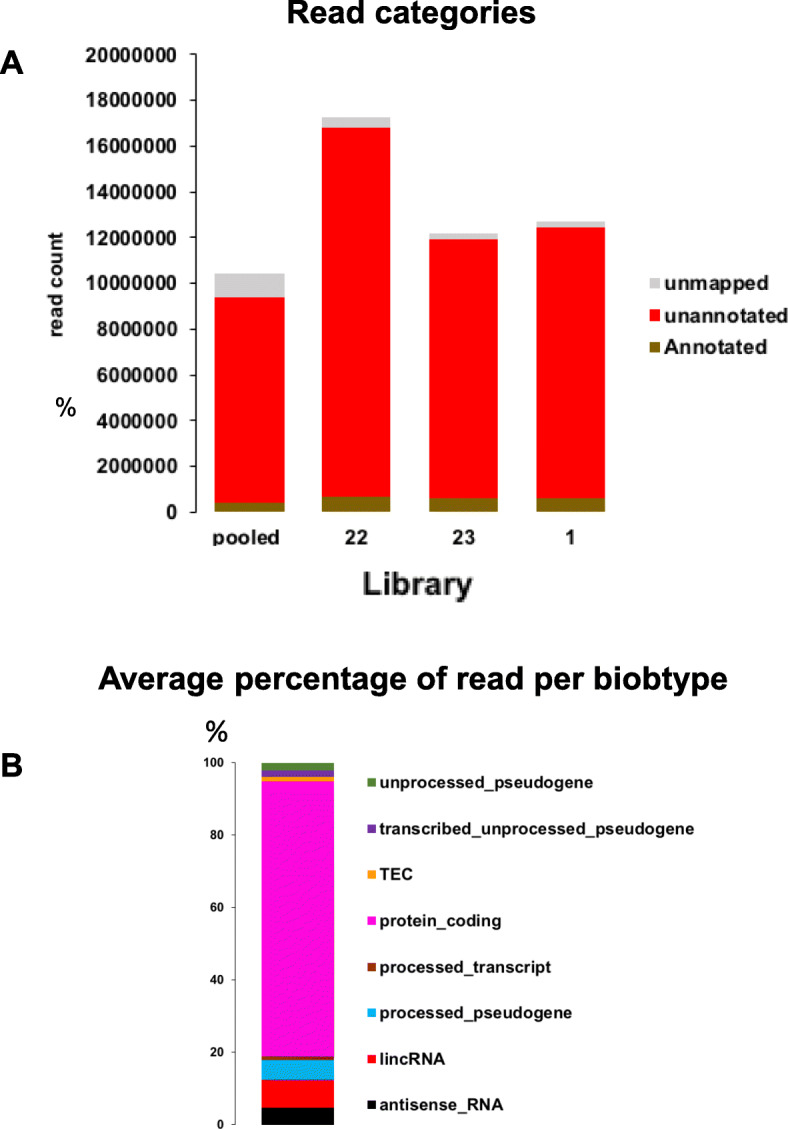
DNA sequencing results. **A**: number of reads per category (annotated, unannotated, unmapped) **B**: percentage of read per biotype

The DNA sequencing reads could be assigned to three categories: annotated, unannotated and unmapped. The pooled sample produced the lowest number of reads (Fig. 3A), consisting of 3 categories: annotated (3,8%), unannotated and unmapped (10%) with the larger number aligning with unannotated regions of the human genome (86,2%). Samples from individuals had very similar distribution of reads: 2,3% of reads not aligning to the human genome and 4,5% aligning to annotated region of the human genome, while the largest category (93,1%) corresponded to unannotated region of the human genome (Fig. 3A). The distribution of annotated reads into different biotypes (Fig. 3B) was similar across all 4 samples, the most abundant being protein coding genes (73–75%), followed by LincRNA (6,5–8%), processed pseudogene (4,5-5,3%) and antisense RNA (4–4,5%). The coverage of the protein coding genes was very small, except for those encoded by the mitochondrial chromosome.

### Sweat particle characterization

**Fig. 4:**
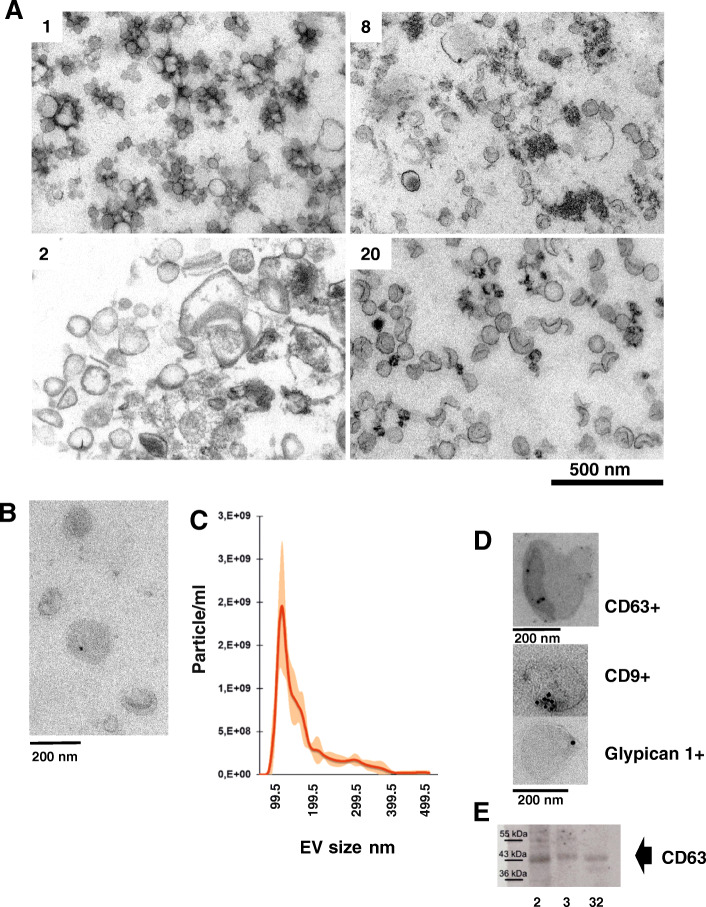
Sweat particles. Visual characterization of sweat particles. Panels **A**: TEM, thin sections of plastic embedded pelleted sweat from 4 donors number corresponding to sequencing library (Table 1). **B**: TEM, negative staining of ExoEasy isolated sweat EVs, **C**: NTA analysis of Exoeasy EVs, **D**: TEM, Immunostaining of isolated sweat EVs, E: western blot, protein from Exoeasy sweat preparations were stained with anti-CD63 antibody (ab193349). Full size western blot with region selected marked is shown as supplementary Fig. 6

The presence of high amount of mitochondrial DNA suggested the presence of organelles in addition to EVs in the samples. To determine if this was the case, thin sections of filtered (0,8 μm before concentration, 0,45 μm after) sweat pellets were made from individually processed samples and analyzed by transmission electron microscopy (TEM) (Fig. 4A). We found vesicular structures of varying sizes and appearances in the individual samples, including some with clear double membranes, indicating presence of EVs. Most vesicles were in the 100 nm range, but some individual samples were richer in smaller and/or larger EVs. We were unable to detect any recognizable mitochondria (Fig. 4A), but bacteria were occasionally detected when 0,45 μm filtration was omitted (data not shown).

We chose ExoRNEasy kit to directly purify RNA from concentrated sweat to capture a more diverse selection of EVs [19]. Concentrated individual sweat samples prepared with ExoEasy had variable amounts of well-defined double membrane EVs of 50 to 200 nm sizes (Fig. 4B, Supplementary Figure 3). Image of negative control (instead of sweat, glove was filled with PBS, which was subsequently processed like volunteer sample) is also shown in Supplemental figure 3. NTA analysis of ExoEasy sweat samples showed 1 peak at around 100 nm and much smaller peaks for 200 and 300 nm (Fig. 4C, supplementary Figure 4). Immuno- transmission electron microscopy detected the presence of typical EV markers: CD63 and CD9 in individual sweat EVs and other markers like Glypican1 (Fig. 4D). Presence of CD63 was confirmed by western blotting, while staining for Argonaute 2 and GM130 were negative (Fig. 4E, Supplementary Figure 5). No CD63 was detected in flowthrough from ExoEasy columns and in negative control from gloves (Supplementary Figure 5). Average particle /ml of sweat was 475,000 but a wide range was observed (35000–1 million particle /ml) with number of particles per μg of protein being in the range of 0,3–6*10^9^ particles/μg protein (*n* = 4). We have submitted all relevant data of our experiments to the EV-TRACK knowledgebase (EV-TRACK ID: EV210083) [20].

### EV-enriched sweat RNA analysis

**Fig. 5:**
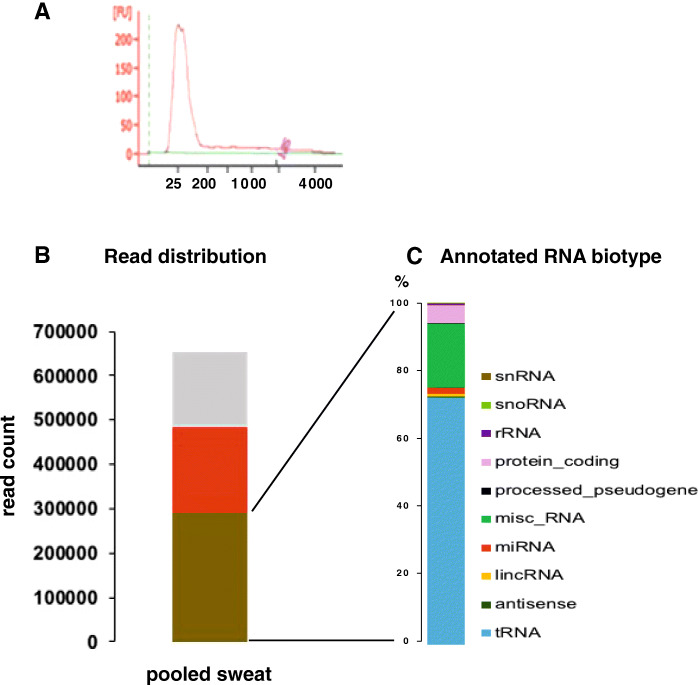
Sweat smallRNA-seq from pooled sample. **A**: bioanalyzer profile of sweat Exoeasy RNA, **B**: read distribution, **C**: biotype distribution of annotated reads

We used the remaining ultracentrifugation pellets from pooled sweat to extract RNA from EV-enriched sweat fraction using ExoRNEasy kit (Fig. 1). Profiling of extracted RNA on bioanalyzer picoChip (Agilent) showed only small RNA with sizes ranging from 20 to 200 bp with no obvious 18 or 28 s ribosomal RNA (Fig. 5A), subsequently Small RNA protocol was used for the sequencing on Ion Torrent PGM (Thermo Fisher Scientific). A total of 652,280 trimmed reads were used for alignment to the human genome using Bowtie 1. Reads fell into 3 categories: annotated (44,6%, tRNA reads were included in this category), unannotated 29,6% and unmapped 24,7% (Fig. 5B). Over 70% of the annotated human reads were identified as tRNA, 18,5% as miscRNA, 5% mRNA and 1,85% miRNA (Fig. 5C).

#### miRNA from pooled EV-enriched sweat

**Fig. 6:**
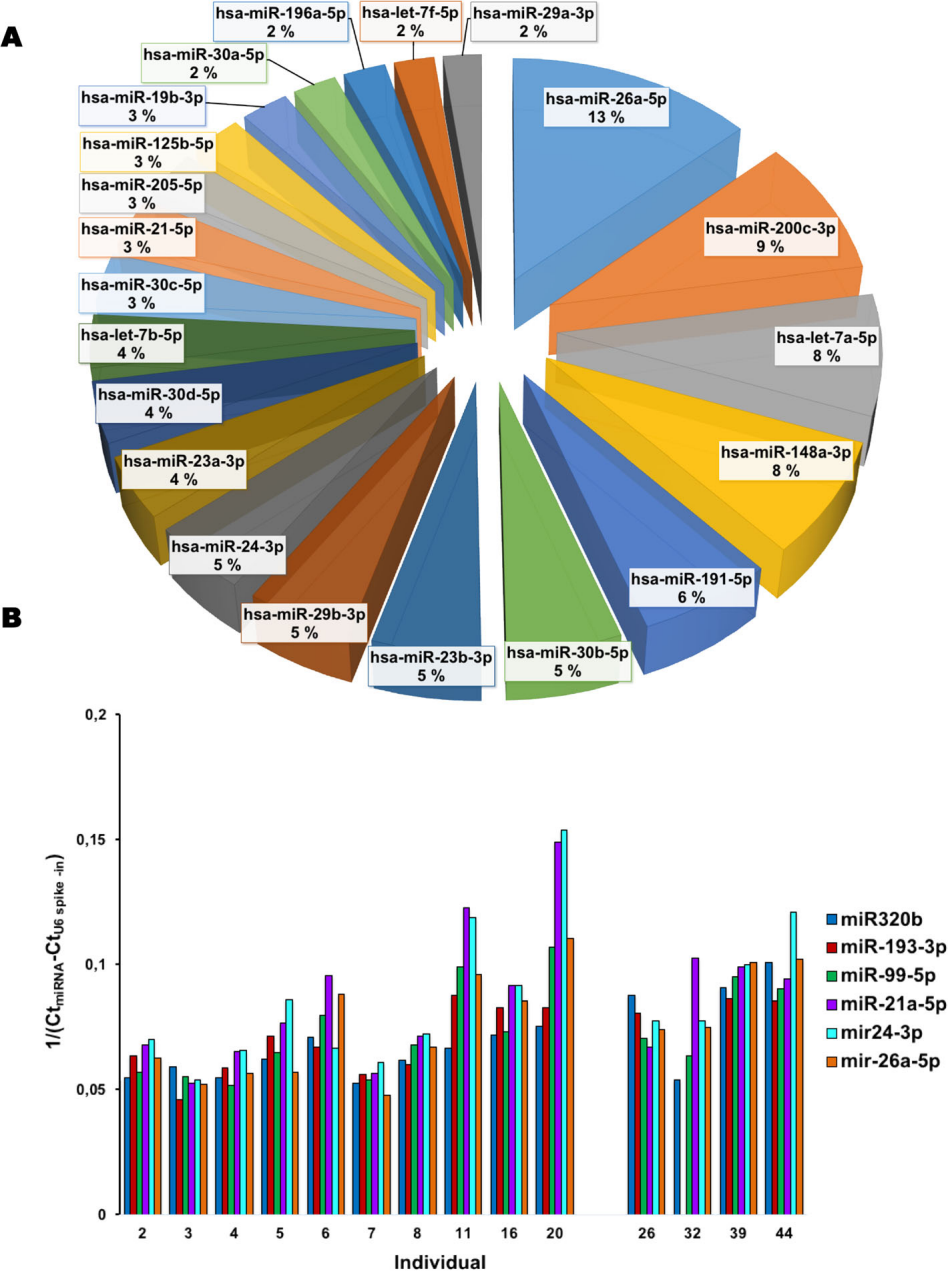
EV-enriched sweat /associated miRNA. **A**: most represented miRNA (minimum read count > 100) in pooled sweat sample. **B**: Comparison of specific miRNA level in individual samples. Last 4 samples were not sequenced

66 miRNAs with read count 10 or higher were identified (Table 2). miR26a-5p was the miRNA with the most reads, followed by miR200c-3p, miRLet7A and miR148a-3p (Fig. 6A). We selected 6 miRNAs for testing by qPCR from most abundant (miR26a-5p/692 reads) to low (miR320b/10 reads) on 14 individual samples of sweat RNA (10 were subsequently used for RNA sequencing and 4 additional ones were not, Table 1) and compared their level relative to each other, inside each sample. All the miRNAs were detected in all the samples except one, where miR193–3p, was undetectable. In most cases miR21 -5p and miR24-3p were the highest, not miR26a-5p (Fig. 6B).

**Table 2:**
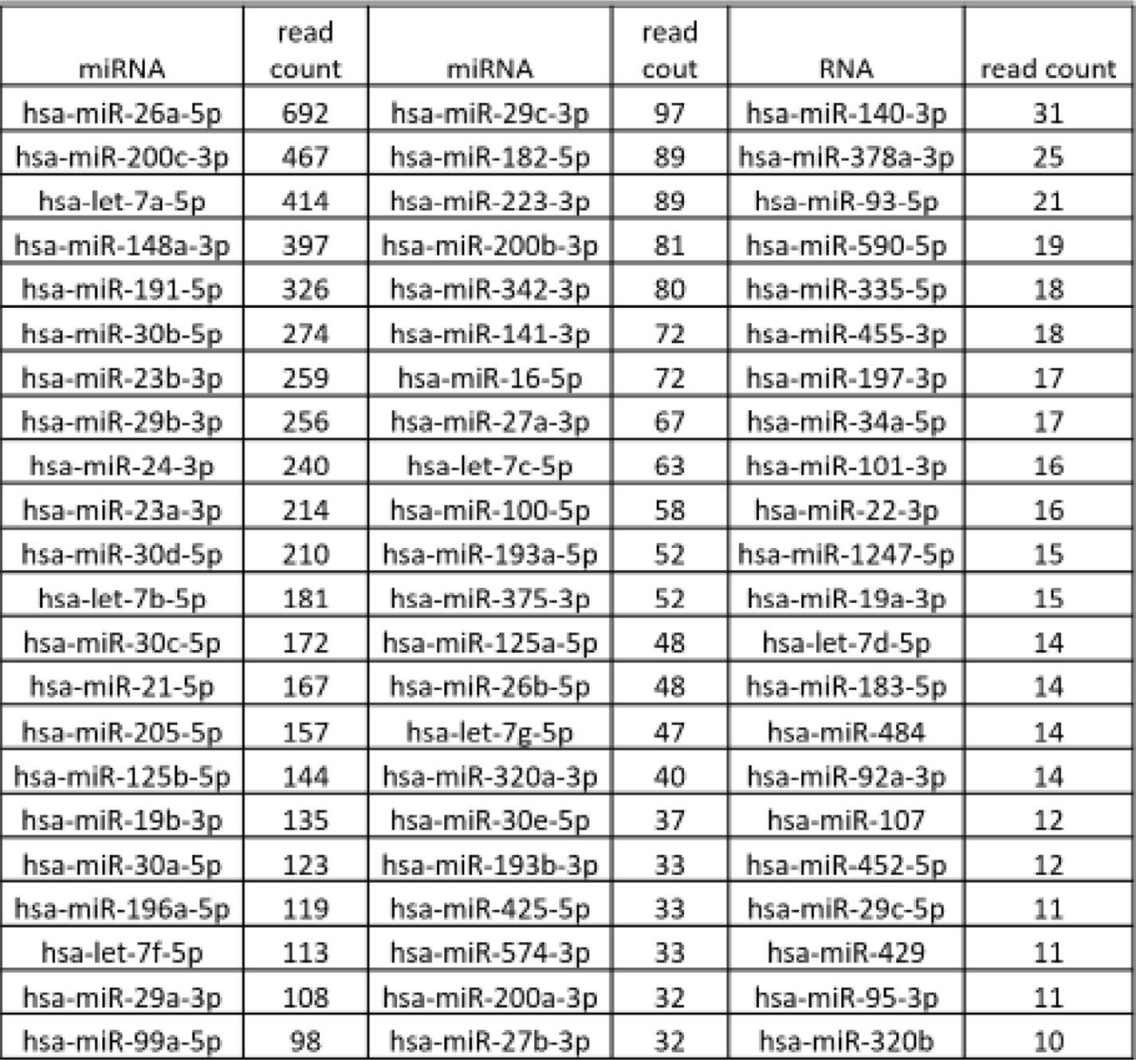
Sweat miRNA pooled samples. miRNA with read count 10 or above

#### RNA-seq from individual volunteers

We then prepared RNA from individual sweat collections, from 6 females and 14 males (Table 1) replacing ultracentrifugation by concentration with Centricon Plus-70 columns (Millipore) with a 100 K kDa cut-off. Bioanalyzer (Agilent) RNA profiles of all samples were similar to each other but yields were highly variable (supplementary Figure 1) and below what can be accurately quantified. We selected higher size fragments (145-200 bp) than recommended by the library kit manufacturer (New England Biolab) to limit the number of empty reads and characterize larger RNA species, including protein coding RNA, as a result very few miRNA reads were identified.

**Fig. 7:**
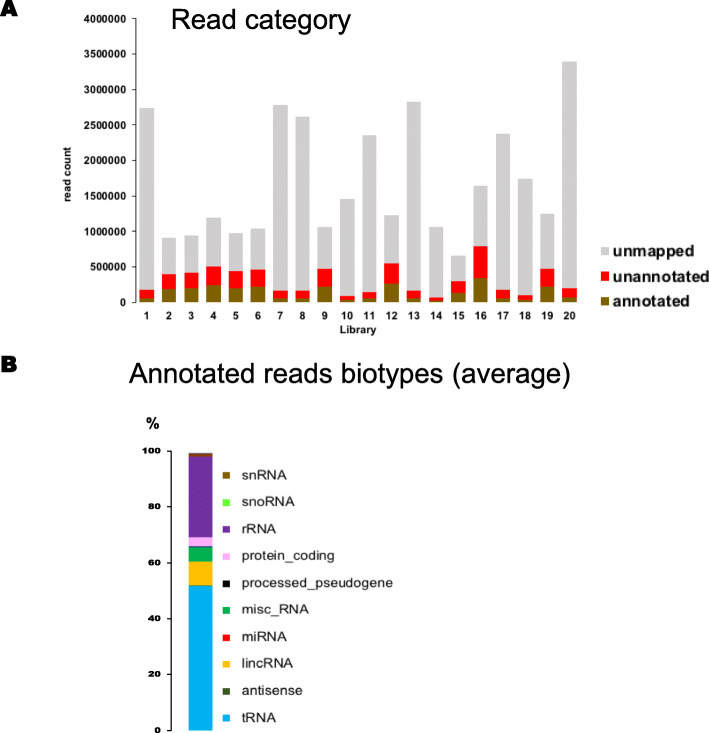
RNA-seq from individual EV-enriched sweat sample. **A**: Distribution of reads from each library in categories, **B**: distribution of annotated reads in biotypes (average from all 20 samples)

**Fig. 8:**
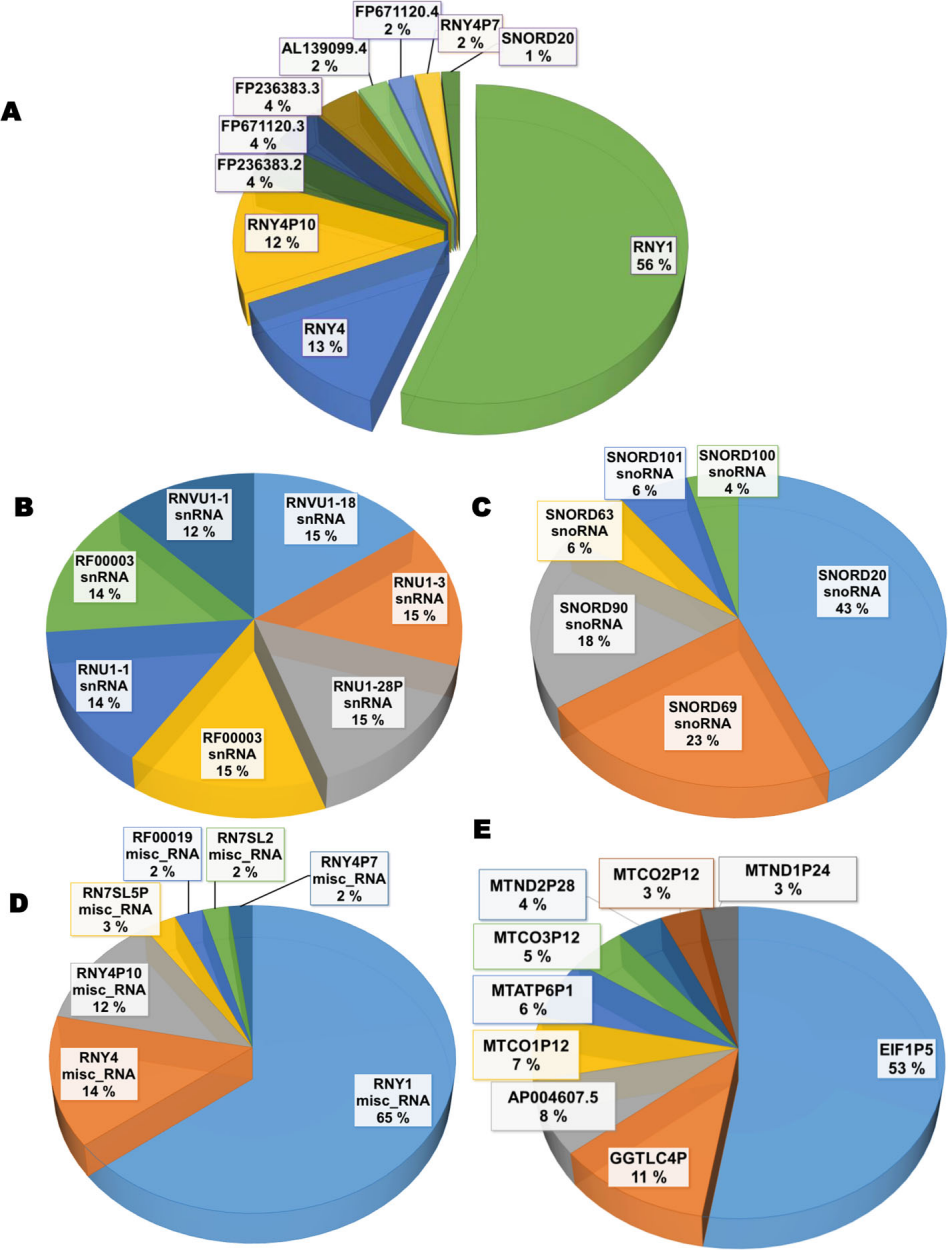
Distribution of RNA biotypes (pooled data). **A**: Data were pooled for analysis most abundant RNAs (tRNA and rRNA we removed from analysis), **B**: most abundant SnRNA subtypes, **C**: most abundant SnoRNAs, **D**: most abundant misc_RNA, E: most abundant unprocessed pseudogenes

After quality trimming, number of reads per sample ranged from 650,000 to 3,4 million (Fig. 7A) with a high number of unmapped reads. As the number of annotated reads per sample were low, we analyzed them together. The distribution into biotypes showed over 50% identified as tRNA, 28% as rRNA and LincRNA, miscRNA and protein coding between 8 and 3% (Fig. 7B). Excluding tRNA and rRNA the top 10 genes identified include 6 miscRNAs with RNY1, RNY4, and RNY4P10 being the most represented, 3 LincRNA, 1 non-coding RNA, 1 snoRNA (SNORD20) (Fig. 8A). As MIR6087 is no longer considered a miRNA, it was omitted from the figure.

Although the function of small nuclear RNA is to participate in mRNA splicing in the nucleus, these small RNA species are abundant in EV-enriched sweat. In Fig. 8B the seven most abundant snRNAs represent each between 12 and 15% of snRNAs identified, all seven represented in B are detected in at least 19 samples (supplementary Table I) and they belong to U1 and U5 families.

The snoRNA’s main characterized role is the modification of rRNA, 11 of them are found in significant amounts in EV-enriched sweat, the most abundant type found is box C/D, which guides the 2′-O-methylation of rRNA SNORD20 represent over 40% of the total, SNORD90 and SNORD69 (targeting 28 s rRNA) around 20%, SNORD63 and SNORD101 6%, SNORD100 4% (Fig. 8C). SNORD20, SNORD69 and SNORD63 were identified in at least 19 of the 20 samples (Supplementary Table I). Another type of RNA modifying small RNA closely related to snoRNA and located in Cajal bodies (small organelles of the nucleolus of proliferative cells) has 2 well represented members in sweat: scRNA11 and scaRNA4. RNY1 represent over 60% of misc-RNA biotype’s reads, RNY4 represents 16%, RNY4P10 14% and RNY4P7 2% (Fig. 8D).

Unprocessed pseudogenes (Fig. 8E) are created by duplication of existing genes and retain intron-exon structure, in this biotype, EIF1P5 is overrepresented with 53% of the reads mapping to it, while GGTLC4P represent 11% and AP004607.5 8%, the remaining unprocessed pseudogenes are mitochondrial genes inserted in nuclear chromosomes and they represent less than 7% each. Processed pseudogenes, which arise by retrotransposition and are therefore inserted in the genome without intronic sequences were also identified. Top 1% of reads from RNA-seq included 86 processed pseudogenes.

Piwi-interacting RNA (piRNA) are small noncoding RNA first identified in the germline. They are short: 24-30 bp and their first identified function was to silence transposons. They have since been also identified in other cells and body fluids and may have potential as biomarkers. Only a very small percentage of the reads of each sample can be identified as piRNAs, but 5 piRNAs were identified in all 20 samples, and 6 in 19 samples (Supplementary Figure 2). Another 1000 piRNAs were sporadically detected in 3 or less samples. Other non-coding RNA usually associated with EVs like Vault RNA were also identified in the majority of the samples (Supplementary Table 1).

#### Sweat mRNA

**Fig. 9:**
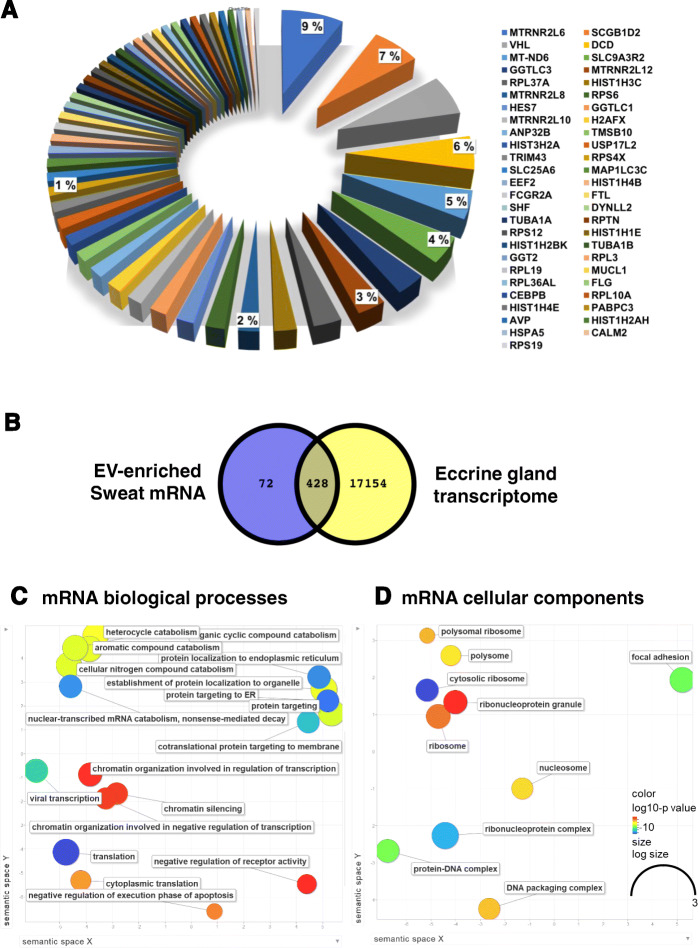
EV-enriched Sweat mRNA (pooled data). **A**: most represented mRNA, **B**: overlap between human eccrine gland transcriptome and EV-enriched sweat mRNA, **C**: enriched biological processes in EV-enriched sweat mRNA, **D**: enriched cellular components, based on GO annotations, colors represent log10 *p*-value representation using Revigo

The most abundant mRNAs in sweat are encoded by the mitochondrial genome, followed by a mitochondrial transcript encoded by a nuclear gene, MTRNR2L6 (Fig. 9A). Comparison with a recent report of the transcriptome and proteome of human eccrine gland shows that 85% of mRNAs found in EV-enriched sweat overlap with mRNA from sweat eccrine gland, with only 14.4% unique to EV-enriched sweat (Fig. 9B).

For enrichment analysis of GO annotation we selected transcript with FPKM values bigger than 25, most of the transcript encode either translation related proteins, nucleic acid binding protein or focal adhesion protein. Biological Processes involve energy metabolism, protein synthesis and nucleic acid binding (Fig. 9C), the most represented cell components are ribosomal (Fig. 9D). In addition to the abundant mRNA species (Table 3), 6675 additional gene products were detectable in 1–3 samples with FPKM value bigger than 0.

**Table 3:**
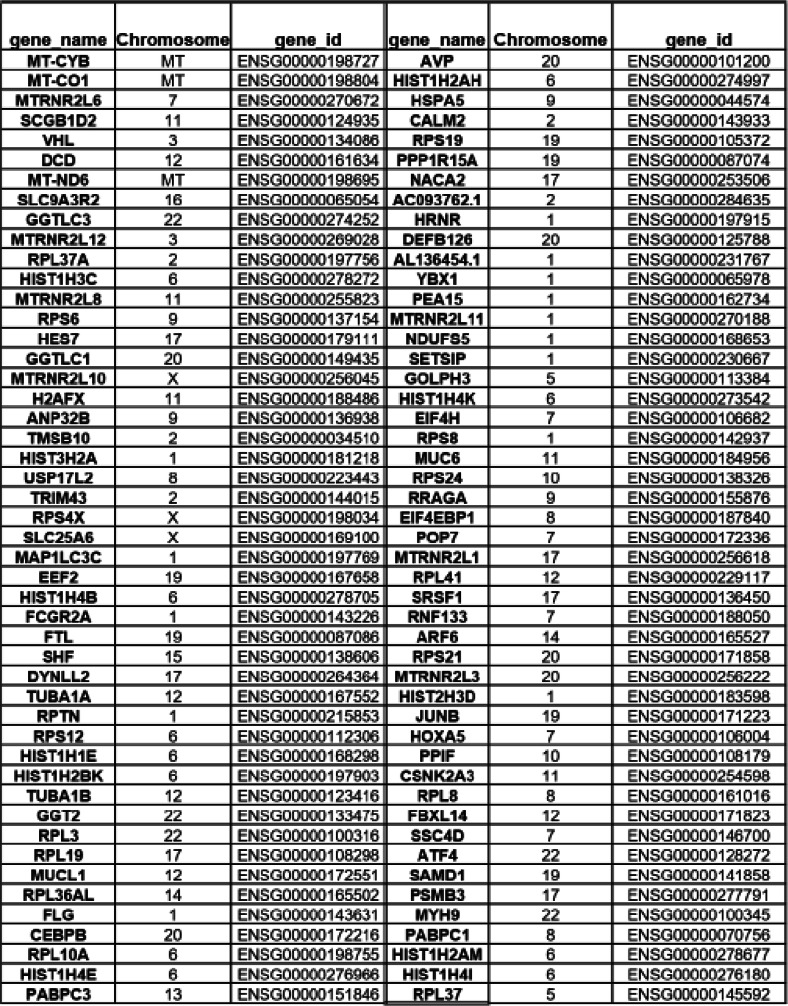
mRNA with highest FPKM IN RNA-SEQ (pooled data from individual). mRNA, including mitochondrial mRNA most represented in sweat, with chromosome location and ensemble gene ID

**Fig. 10:**
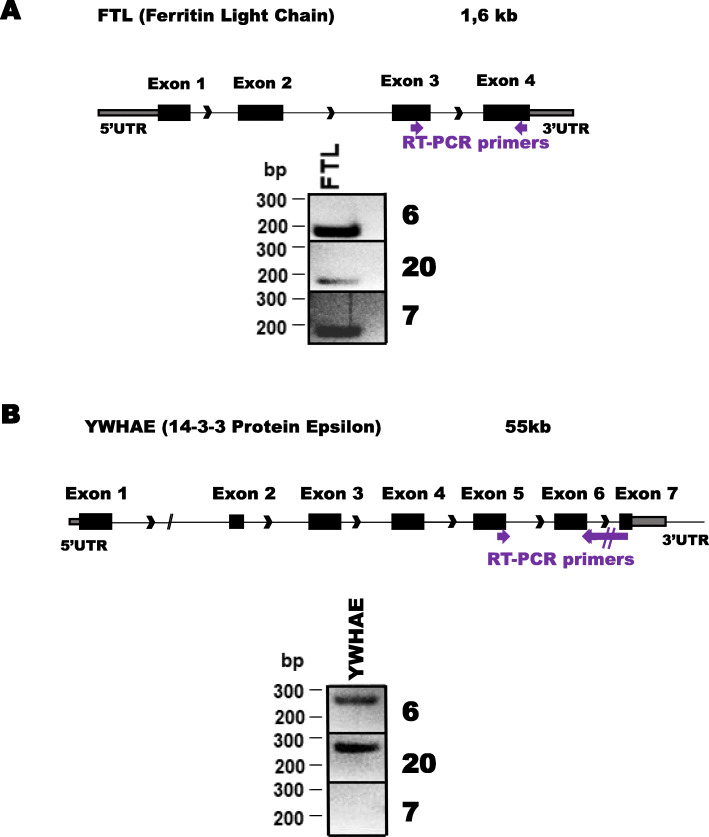
spliced mRNA are detectable in EV-enriched sweat. **A**: Ferritin Light Chain Gene, PCR primers localization (arrows) with RT-PCR amplified DNA visualized on 2% agarose gel, **B**: 14–3-3 Protein Epsilon gene with PCR primers spanning exon5,6 and 7, below product on agarose gels showing amplification across the 3 exons in 2 out of 3 tested samples. The full size gels with region selected marked are shown in supplemental Fig. 7

Because the reads for mRNA could be detected on several exons and alignment with STAR showed that some of these reads were spliced, we checked the presence of spliced mRNA in EV-enriched sweat by RT-PCR with primers designed for amplification across splice junctions. The most abundant and largely distributed in most of the samples was (ferritin light chain) FTL mRNA, FTL gene has 4 exons, and using primers designed to amplify mRNA of the last 2 exons (3 and 4), we were able to amplify cDNA from several samples (Fig. 10A). YWHAE gene spans across 7 exons, on a total DNA length of 55 kb, with a reverse PCR primer spanning exon 6 and 7 together and forward primer on exon 5 we amplified a fragment of the expected size in 2 out of 3 samples (Fig. 10B, supplementary Figure 7). We were able to amplify several other mRNA at least across one splice junction indicating that even if the RNA is fragmented it is processed (supplementary figure 7).

### Metagenomic nucleic acid

#### Microbial DNA and RNA

**Fig. 11:**
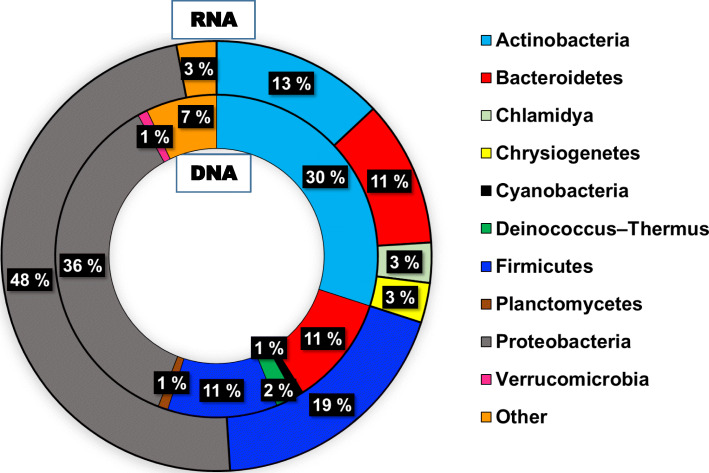
Sweat Metagenomics. Most represented bacterial orders in RNA sequencing (outer circle) and DNA sequencing (inner circle)

DNA-seq had 5–9% of unmapped reads, and to determine their origin, the reads were assembled and aligned against the metagenome and the main taxonomical orders were identified. In addition to bacterial DNA, there was a small proportion of virus and archaeal DNA. Dominant bacterial orders were Proteobacteria, Actinobacteria followed by equal proportion of Bacteroidetes and Firmicutes (Fig. 11), a distribution typical of skin microbiota.

RNA-seq produced much higher amounts of unmapped reads, and metagenomic analysis attributed the highest proportion of them to bacteria, but fungi and virus could also be identified. The distribution of the main bacterial orders was relatively similar to what we observe for DNA, except for a larger proportion of Firmicutes than Bacteroidetes (Fig. 11), again a distribution consistent with the skin microbiome.

**Fig. 12:**
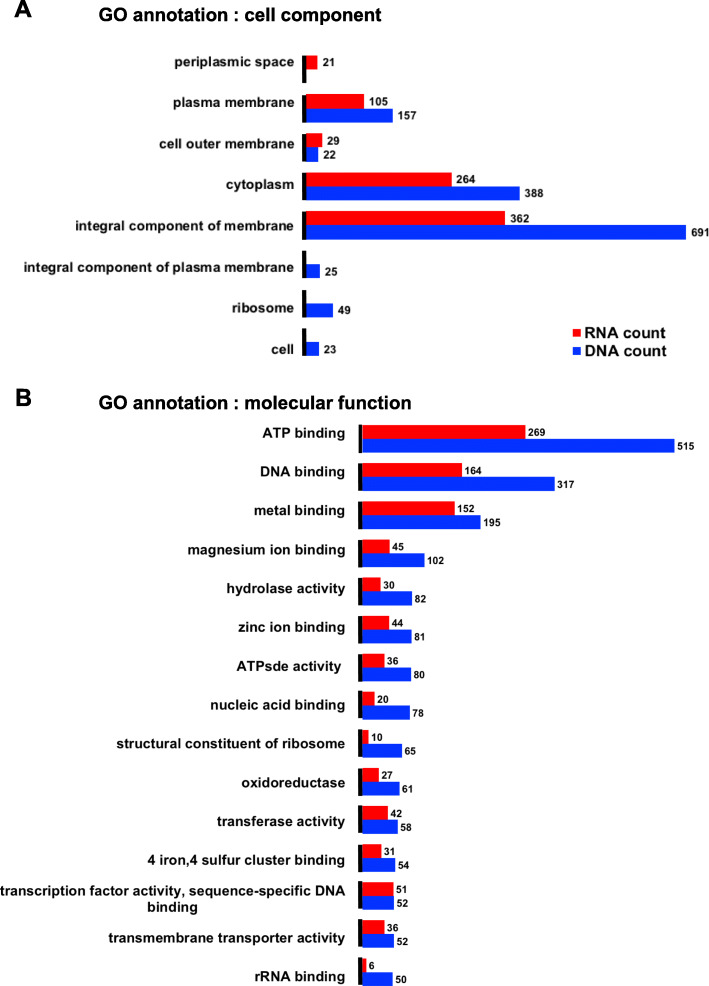
bacterial GO annotations. Most represented GO annotation in bacterial genes identified A: most represented cell component. B: most represented molecular function with total count number indicated

A fraction of the sequences identified corresponded to microbial protein coding genes. We retrieved the protein IDs with GO annotations from UniProt database, then counted the GO annotations. The cell component annotations showed mostly integral components of membrane and cytoplasm for both DNA and RNA sequencing. DNA sequencing included also at least 20 protein coding genes with annotations for cell, ribosome and integral component of plasma membrane, while RNA-seq included periplasmic space linked mRNA (Fig. 12A). The molecular functions of protein coding genes identified in both DNA-seq and RNA-seq were predominantly ATP binding, DNA binding and metal ion binding (Fig. 12B).

#### Viral DNA

We found 2 types of viral DNA, from human virus: papilloma, polyoma, herpes virus and from bacteriophages infecting the bacteria from the skin. Viral sequences represent 13% of the total identified, with papilloma sequences representing 24% of viral sequences, polyomavirus represent 5% and herpes 2%. Only a portion of the DNA identified encodes identified protein, but from papillomavirus 6 protein coding genes can be identified (out of a total of 8), capsid protein major L1 and Minor L2, replication protein E1, regulatory protein E2, and protein E4 and E6. From polyoma viruses, and Merkel cell polyoma virus, Small T antigen and capsid protein Vp1 (total encoded by viral genome: 5–9 protein) were identified and from human Cytomegalovirus, only uncharacterized protein UL126 (more than 165 protein coding genes). The gene encoded are capsid protein, regulatory and replication protein from papilloma virus and capsid and small T antigen from polyomavirus (Table 4).

**Table 4:** Human virus genes identified in sweat DNA

Protein names	Gene names	Organism	Length
Major capsid protein L1	L1	Gammapapillomavirus 9	521
Major capsid protein L1	L1 gp7	Human papillomavirus type 209	507
Major capsid protein L1	L1	Human papillomavirus	514
Major capsid protein L1	L1	Gammapapillomavirus 9	513
Major capsid protein L1	L1	Human papillomavirus 204	508
Major capsid protein L1	L1	Human papillomavirus 202	528
Major capsid protein L1	L1	Human papillomavirus type 200	514
Major capsid protein L1	L1	Human papillomavirus type 49	509
Major capsid protein L1	L1	Gammapapillomavirus 22	517
Major capsid protein L1	L1	Gammapapillomavirus 12	507
Major capsid protein L1	L1	Human papillomavirus type 94	532
Major capsid protein L1	L1	Human papillomavirus type 8	514
Major capsid protein L1	L1	Human papillomavirus type 48	513
Major capsid protein L1	L1	Human papillomavirus 110	506
Major capsid protein L1	L1	Betapapillomavirus 2	508
Major capsid protein L1	L1	Human papillomavirus 174	507
Major capsid protein L1	L1	Human papillomavirus type 168	523
Major capsid protein L1	L1	Gammapapillomavirus 16	516
Major capsid protein L1	L1	Human papillomavirus type 137	516
Major capsid protein L1	L1	Gammapapillomavirus sp.	517
Major capsid protein L1	L1	Human papillomavirus 138	514
Major capsid protein L1	L1	Human papillomavirus type 37	507
Major capsid protein L1	L1	Human papillomavirus type 94	532
Minor capsid protein L2	L2	Human papillomavirus 120	519
Minor capsid protein L2	L2	Betapapillomavirus 1	520
Minor capsid protein L2	L2	Human papillomavirus type 168	520
Minor capsid protein L2	L2	Gammapapillomavirus 16	507
Minor capsid protein L2	L2	Human papillomavirus 202	498
Minor capsid protein L2	L2	Human papillomavirus type 94	459
Minor capsid protein L2	L2	Betapapillomavirus 2	522
Minor capsid protein L2	L2	Human papillomavirus type 195	516
Minor capsid protein L2	L2	Human papillomavirus type 37	534
Minor capsid protein L2	L2	Gammapapillomavirus sp.	518
Minor capsid protein L2	L2	Gammapapillomavirus 24	525
Minor capsid protein L2	L2	Human papillomavirus 204	503
Minor capsid protein L2	L2	uncultured Papillomavirus	521
Minor capsid protein L2	L2	Gammapapillomavirus 12	521
Minor capsid protein L2	L2	Gammapapillomavirus 22	517
Minor capsid protein L2	L2	Human papillomavirus	510
Minor capsid protein L2	L2	Human papillomavirus type 200	502
Minor capsid protein L2	L2	Betapapillomavirus 2	529
Minor capsid protein L2	L2	Human papillomavirus type 48	502
Minor capsid protein L2	L2	Human papillomavirus 110	537
Minor capsid protein L2	L2 gp6	Human papillomavirus type 209	519
Minor capsid protein L2	L2	Gammapapillomavirus 9	506
Minor capsid protein L2	L2	Human papillomavirus type 8	518
Protein E6	E6	Human papillomavirus type 94	148
Protein E6	E6	Human papillomavirus type 168	139
Protein E6	E6	Human papillomavirus type 8	155
Protein E6	E6	Human papillomavirus 202	143
Protein E6	E6	Human papillomavirus	140
Protein E6	E6	Betapapillomavirus 2	141
Protein E6	E6	Human papillomavirus type 94	148
Protein E6	E6	Human papillomavirus type 137	142
Regulatory protein E2	E2	Human papillomavirus 157	400
Regulatory protein E2	E2 HpV115gp4	Human papillomavirus type 115	481
Regulatory protein E2	E2	Human papillomavirus type 94	378
Regulatory protein E2	E2	Human papillomavirus 204	388
Regulatory protein E2	E2	Human papillomavirus KC5	395
Regulatory protein E2	E2	Human papillomavirus 110	454
Regulatory protein E2	E2	Betapapillomavirus 2	459
Regulatory protein E2	E2	Human papillomavirus type 200	401
Regulatory protein E2	E2	Human papillomavirus	398
Regulatory protein E2	E2	Human papillomavirus type 48	396
E4	E4	Human papillomavirus type 168	160
Replication protein E1 (EC 3.6.4.12) (ATP-dependent helicase E1)	E1	Human papillomavirus 202	605
Replication protein E1 (EC 3.6.4.12) (ATP-dependent helicase E1)	E1	Gammapapillomavirus 22	601
Replication protein E1 (EC 3.6.4.12) (ATP-dependent helicase E1)	E1	Human papillomavirus 157	601
Replication protein E1 (EC 3.6.4.12) (ATP-dependent helicase E1)	E1	Betapapillomavirus 1	620
Replication protein E1 (EC 3.6.4.12) (ATP-dependent helicase E1)	E1	Human papillomavirus type 94	681
Replication protein E1 (EC 3.6.4.12) (ATP-dependent helicase E1)	E1	Gammapapillomavirus 9	600
Replication protein E1 (EC 3.6.4.12) (ATP-dependent helicase E1)	E1	Gammapapillomavirus sp.	610
Replication protein E1 (EC 3.6.4.12) (ATP-dependent helicase E1)	E1	Gammapapillomavirus sp.	604
Replication protein E1 (EC 3.6.4.12) (ATP-dependent helicase E1)	E1	Betapapillomavirus 2	605
Replication protein E1 (EC 3.6.4.12) (ATP-dependent helicase E1)	E1	Human papillomavirus type 48	593
Replication protein E1 (EC 3.6.4.12) (ATP-dependent helicase E1)	E1	Human papillomavirus type 200	598
Replication protein E1 (EC 3.6.4.12) (ATP-dependent helicase E1)	E1	Human papillomavirus type 23	607
Replication protein E1 (EC 3.6.4.12) (ATP-dependent helicase E1)	E1	Human papillomavirus 138	616
Replication protein E1 (EC 3.6.4.12) (ATP-dependent helicase E1)	E1	Human papillomavirus 204	615
Replication protein E1 (EC 3.6.4.12) (ATP-dependent helicase E1)	E1	Betapapillomavirus 2	605
Replication protein E1 (EC 3.6.4.12) (ATP-dependent helicase E1)	E1	Human papillomavirus	601
Replication protein E1 (EC 3.6.4.12) (ATP-dependent helicase E1)	E1	Human papillomavirus type 22	608
Replication protein E1 (EC 3.6.4.12) (ATP-dependent helicase E1)	E1	Human papillomavirus type 168	600
Replication protein E1 (EC 3.6.4.12) (ATP-dependent helicase E1)	E1	Human papillomavirus 116	602
Replication protein E1 (EC 3.6.4.12) (ATP-dependent helicase E1)	E1 gp3	Human papillomavirus type 209	607
Replication protein E1 (EC 3.6.4.12) (ATP-dependent helicase E1)	E1	Human papillomavirus type 137	610
Replication protein E1 (EC 3.6.4.12) (ATP-dependent helicase E1)	E1	Human papillomavirus	600
Replication protein E1 (EC 3.6.4.12) (ATP-dependent helicase E1)	E1	Human papillomavirus type 49	609
Replication protein E1 (EC 3.6.4.12) (ATP-dependent helicase E1)	E1	Human papillomavirus type 37	609
Replication protein E1 (EC 3.6.4.12) (ATP-dependent helicase E1)	E1	Human papillomavirus	610
Small T antigen		MW polyomavirus	206
Small T antigen		Merkel cell polyomavirus	186
ST (Small T antigen)		Human polyomavirus 7	193
Capsid protein VP1	VP1	MW polyomavirus	403
VP1	VP1	Human polyomavirus 7	380
Uncharacterized protein UL126	UL126	Human cytomegalovirus (strain AD169) (HHV-5) (Human herpesvirus 5)	134

Bacterial phages are represented by a variety of genes, 96 are uncharacterized, 274 genes encode phage structural protein and enzymes (Table 5), which molecular functions include mainly DNA binding, helicase, hydrolase and endonuclease. Most components of the phage genome are represented (Table 5).

**Table 5:** Bacteriophages identified in sweat DNA

Protein names	Gene names	Organism	Length
aGPT-Pplase2 domain-containing protein	3 ZEMANAR_3	Mycobacterium phage Zemanar	324
Amidase	ami	Propionibacterium phage PAS10	287
AP2/ERF domain-containing protein	AB9_137	Acinetobacter phage vB_AbaM_B9	262
ATP-dependent helicase	71 SEA_CATERPILLAR_71	Arthrobacter phage Caterpillar	411
ATP-dependent RNA helicase		Pseudoalteromonas phage H103	599
ATPase_AAA_core domain-containing protein	BCP78_0083	Bacillus phage BCP78	358
Baseplate J-like protein	39 SEA_COLUCCI_39	Arthrobacter phage Colucci	373
Beta_helix domain-containing protein	Eldridge_088	Bacillus phage Eldridge	510
Capsid & capsid maturation protease	13 SEA_CATERPILLAR_13	Arthrobacter phage Caterpillar	717
Capsid and capsid maturation protease	13 SEA_MEDIUMFRY_13	Arthrobacter phage MediumFry	717
Capsid and scaffold protein		Propionibacterium phage PA1–14	186
Capsid maturation protease	5 SEA_COLUCCI_5	Arthrobacter phage Colucci	649
Capsid maturation protease	SEA_C3PO_14	Corynebacterium phage C3PO	442
Capsid protein		Staphylococcus phage phiIPLA-C1C	291
Cas4 family exonuclease	SEA_NATOSALEDA_55	Rhodococcus phage Natosaleda	268
CMP deaminase	SEA_WEASELS2_199	Rhodococcus phage Weasels2	118
Collagen-like protein	PHL308M00_19	Propionibacterium phage PHL308M00	268
Collagen-like protein	PHL150M00_19	Propionibacterium phage PHL150M00	268
DNA encapsidation protein	P9AB12kb_p002	Pectobacterium phage DU_PP_III	363
DNA helicase	52 SEA_HOTFRIES_52	Streptomyces phage HotFries	390
DNA helicase	93 PBI_COUNT_93	Microbacterium phage Count	435
DNA helicase	SEA_LUCKYBARNES_64	Brevibacterium phage LuckyBarnes	445
DNA helicase	SEA_MEAK_33	Propionibacterium phage MEAK	317
DNA methylase	65 SEA_MOOMOO_65	Mycobacterium phage MooMoo	542
DNA methylase	61 SEA_NERUJAY_61	Mycobacterium phage Nerujay	365
DNA methylase	SLPG_00003	Salicola phage CGphi29	328
DNA methylase	FLORINDA_85	Mycobacterium phage Florinda	482
DNA methylase	43 GALAXY_43	Arthrobacter phage Galaxy	439
DNA methylase	SEA_YASSJOHNNY_96	Mycobacterium phage YassJohnny	187
DNA methylase	SEA_MURICA_102	Mycobacterium phage Murica	602
DNA methylase	61 PBI_SMEAGOL_61	Mycobacterium phage Smeagol	356
DNA methylase	60 PBI_MUSEUM_60	Mycobacterium virus Museum	465
DNA polymerae/primase	NIKTSON_56	Arthrobacter phage Niktson	1314
DNA polymerase	P9AB12kb_p001	Pectobacterium phage DU_PP_III	690
DNA polymerase I	SEA_LUCKYBARNES_45	Brevibacterium phage LuckyBarnes	621
DNA polymerase III alpha subunit	SEA_DARWIN_47	Corynebacterium phage Darwin	1097
DNA polymerase III alpha subunit	SEA_C3PO_43	Corynebacterium phage C3PO	1097
DNA polymerase/primase	54 SEA_CATERPILLAR_54	Arthrobacter phage Caterpillar	1309
DNA primase	SEA_LUCKYBARNES_63	Brevibacterium phage LuckyBarnes	804
DNA primase	31 P141_31	Propionibacterium phage P14	133
DNA primase	Salvo_71	Xylella phage Salvo	833
DNA primase	Iz_58	Brucella phage Iz	496
DNA primase/polymerase	SEA_C3PO_38	Corynebacterium phage C3PO	847
DNA primase/polymerase	58 SEA_NIGHTMARE_58	Arthrobacter phage Nightmare	1312
DNA single strand annealing protein Erf	uvFWCGRAMDCOMC203_065	Freshwater phage uvFW-CGR-AMD-COM-C203	226
Endolysin	20 P11_20	Propionibacterium phage P1.1	284
Endonuclease	45 SEA_THESTRAL_45	Streptomyces phage Thestral	400
Endonuclease VII	18 SEA_PHISTORY_18	Gordonia phage Phistory	342
Exonuclease	WIZZO_26	Propionibacterium phage Wizzo	348
Exonuclease	MRAK_36	Propionibacterium phage MrAK	313
Exonuclease		Pseudoalteromonas phage H103	292
Gp008	Pepy6gene008	Rhodococcus phage ReqiPepy6	118
Gp067	Pepy6gene067	Rhodococcus phage ReqiPepy6	226
Gp069	Poco6gene069	Rhodococcus phage ReqiPoco6	297
Gp077	Pepy6gene077	Rhodococcus phage ReqiPepy6	193
Gp14	PaP-PAS50_gp14	Propionibacterium phage PAS50	921
Gp16		Propionibacterium phage PA6	385
Gp48	PaP-PAD20_gp48	Propionibacterium phage PAD20	100
H_lectin domain-containing protein	PHL055N00_17	Propionibacterium phage PHL055N00	276
Head protein		Actinomyces virus Av1	455
Head-to-tail adaptor	14 SEA_KYKAR_14	Mycobacterium phage Kykar	125
Head-to-tail connector	12 BARRETLEMON_12	Arthrobacter phage BarretLemon	155
Head-to-tail connector protein	SEA_LILBANDIT_8	Propionibacterium phage LilBandit	115
Helix-turn-helix DNA binding domain protein	132 PBI_COUNT_132	Microbacterium phage Count	927
Helix-turn-helix DNA binding domain protein	78 SEA_LIBERTYBELL_78	Streptomyces phage LibertyBell	910
Helix-turn-helix DNA binding domain protein	PROCRASS1_25	Propionibacterium phage Procrass1	106
Helix-turn-helix DNA binding domain protein	76 PBI_CAMILLE_76	Microbacterium phage Camille	925
Helix-turn-helix DNA binding domain protein	90 SEA_RAINYDAI_90	Streptomyces phage Rainydai	891
Helix-turn-helix DNA binding protein	94 SEA_KEANEYLIN_94	Arthrobacter phage KeaneyLin	891
HNH endonuclease	SEA_SCAP1_2	Streptomyces phage Scap1	135
HNH endonuclease	SEA_ATTOOMI_53	Streptomyces phage Attoomi	101
HNH endonuclease	SKKY_47	Propionibacterium phage SKKY	100
HNH endonuclease	65 SEA_PHAYONCE_65	Mycobacterium phage Phayonce	196
HNH endonuclease		Rhodococcus phage RRH1	91
HNH homing endonuclease		Staphylococcus phage phiIPLA-C1C	269
Holin	MRAK_21	Propionibacterium phage MrAK	133
Homing HNH endonuclase	endo IDF_12	Enterococcus phage Idefix	167
HTH DNA binding protein	58 SEA_BARTHOLOMEW_58	Mycobacterium phage Bartholomew	331
J domain-containing protein	75 SEA_FINCH_75	Rhodococcus phage Finch	194
Lower collar protein		Staphylococcus phage St 134	282
LysM domain protein	18 JAWNSKI_18	Arthrobacter phage Jawnski	221
Major capsid protein	9 MARTHA_9	Arthrobacter phage Martha	295
Major capsid protein	gp79 E3_0790	Rhodococcus phage E3	333
Major capsid subunit	8 JAWNSKI_8	Arthrobacter phage Jawnski	297
Major head protein	PHL141N00_06	Propionibacterium phage PHL141N00	315
Major head protein	mjh	Propionibacterium phage PAD21	314
Major head protein	PHL082M00_06	Propionibacterium phage PHL082M00	323
Major tail protein	16 GORDON_16	Arthrobacter phage Gordon	290
Major tail protein	SEA_DRPARKER_11	Propionibacterium phage DrParker	213
Major tail protein	14 SEA_RAINYDAI_14	Streptomyces phage Rainydai	294
Major tail protein	LAUCHELLY_11	Propionibacterium phage Lauchelly	212
Major tail protein	SEA_C3PO_23	Corynebacterium phage C3PO	220
Major tail sheath	18 PRINCESSTRINA_18	Arthrobacter phage PrincessTrina	482
MazG-like nucleotide pyrophosphohydrolase	41 PBI_PAJAZA_41	Microbacterium phage Pajaza	249
Membrane protein	7 PBI_HYPERION_7	Microbacterium phage Hyperion	238
Membrane protein	26 PBI_POUSHOU_26	Corynebacterium phage Poushou	152
Minor tail protein	SEA_SUPERNOVA_15	Propionibacterium phage Supernova	313
Minor tail protein	SEA_FRANZY_22	Arthrobacter phage Franzy	618
Minor tail protein	SEA_TIMINATOR_21	Arthrobacter phage Timinator	446
Minor tail protein	SEA_AQUARIUS_17	Propionibacterium phage Aquarius	272
Minor tail protein	MRAK_17	Propionibacterium phage MrAK	272
Minor tail protein	SEA_QUEENBEY_16	Propionibacterium phage QueenBey	385
Minor tail subunit	PHL301M00_15	Propionibacterium phage PHL301M00	322
N-acetylmuramoyl-L-alanine amidase domain-containing protein		Propionibacterium phage pa33	286
N-acetylmuramoyl-L-alanine amidase domain-containing protein		Propionibacterium phage pa28	285
Nuclease	SEA_LUCKYBARNES_47	Brevibacterium phage LuckyBarnes	400
p55.1		Xanthomonas virus Xop411	189
PDDEXK_1 domain-containing protein	GMA2_62	Gordonia phage GMA2	331
PDDEXK_1 domain-containing protein	36 P101A_36	Propionibacterium phage P101A	315
Pentapeptide repeat protein	SEP1_136	Staphylococcus phage phiIBB-SEP1	209
Peptidoglycan hydrolase	SEA_BRENT_19	Arthrobacter phage Brent	448
phage terminase, large subunit	g04	Yersinia phage fEV-1	462
POLAc domain-containing protein	GMA2_66	Gordonia phage GMA2	594
Portal	3 P141_3	Propionibacterium phage P14	441
Portal protein	SEA_DRGREY_12	Streptomyces phage DrGrey	450
Portal protein	4 SEA_COLUCCI_4	Arthrobacter phage Colucci	476
Portal protein	KEIKI_3	Propionibacterium phage Keiki	441
Portal protein	SEA_DRPARKER_3	Propionibacterium phage DrParker	441
Portal protein	PHL092M00_03	Propionibacterium phage PHL092M00	441
Portal protein	ArV1_002	Arthrobacter phage vB_ArtM-ArV1	476
Prim-Pol domain-containing protein		Pseudoalteromonas phage H103	761
Putative amidase	PHL060L00_20	Propionibacterium phage PHL060L00	288
Putative bifunctional DNA primase/polymerase	M22_064	Idiomarinaceae phage Phi1M2–2	754
Putative bifunctional DNA primase/polymerase	S708_57	Brucella phage S708	780
Putative capsid	6 P1001_6	Propionibacterium phage P100_1	314
Putative dCTP deaminase	PhAPEC7_24	Escherichia phage vB_EcoP_PhAPEC7	168
Putative DNA helicase	GMA2_64	Gordonia phage GMA2	654
Putative DNA helicase	PHL111M01_33	Propionibacterium phage PHL111M01	317
Putative DNA helicase	PAC5_34	Propionibacterium phage PAC5	287
Putative DNA methyltransferase	55 BRUJITA_55	Mycobacterium virus Brujita	216
Putative DNA primase		Propionibacterium phage PacnesP1	241
Putative DNA primase	PHL111M01_30	Propionibacterium phage PHL111M01	223
Putative DNA primase	PHL085N00_30	Propionibacterium phage PHL085N00	241
Putative DNA primase	PHL111M01_31	Propionibacterium phage PHL111M01	133
Putative endolysin	PHL179M00_20	Propionibacterium phage PHL179M00	296
Putative exonuclease	7S3_41	uncultured Caudovirales phage	281
Putative helicase	Tb_ORF45	Brucella phage Tb	577
Putative major head protein	PHL037M02_06	Propionibacterium phage PHL037M02	316
Putative major tail protein	GMA2_25	Gordonia phage GMA2	139
Putative membrane protein	Twillingate_011	Staphylococcus phage Twillingate	41
Putative phosphoribosyl-ATP pyrophosphohydrolase	SmphiM6_41	Sinorhizobium phage phiM6	129
Putative portal	3 P100D_3	Propionibacterium phage P100D	441
Putative portal protein	PAC4_3	Propionibacterium phage PAC4	406
Putative protease	PHL025M00_16	Propionibacterium phage PHL025M00	385
Putative protease	PHL082M03_16	Propionibacterium phage PHL082M03	385
Putative recA-like NTPase	vBEcoSSa179w3YLVW_00039	Escherichia phage vB_EcoS Sa179lw	274
Putative recA-like NTPase	Sf11_gp7	Shigella phage Sf11 SMD-2017	276
Putative sigma factor	PHL082M03_23	Propionibacterium phage PHL082M03	130
Putative structural protein	GMA2_16	Gordonia phage GMA2	554
Putative structural protein	GMA2_9	Gordonia phage GMA2	584
Putative tape measure	14 P104A_14	Propionibacterium phage P104A	921
Putative tape measure	14 ATCC29399BT_14	Propionibacterium phage ATCC29399B_T	921
Putative tape measure protein	PHL112N00_14	Propionibacterium phage PHL112N00	921
Putative terminase	GMA2_1	Gordonia phage GMA2	559
Putative terminase	PHL111M01_02	Propionibacterium phage PHL111M01	503
Putative terminase large subunit	2 P104A_2	Propionibacterium phage P104A	503
Putative terminase large subunit	ABP12_00064	Acinetobacter phage WCHABP12	433
Putative type III restriction endonuclease	p11sa141_49	Brucella phage 11sa_141	141
Putative VRR-DNA nuclease	M22_057	Idiomarinaceae phage Phi1M2–2	136
Twillingate_149		Staphylococcus phage Twillingate	409
Ribonucleoside-diphosphate reductase large subunit (EC 1.17.4.1)	vBPaeSS218_00016	Pseudomonas phage vB_PaeS_S218	607
Ribonucleotide reductase	SEA_C3PO_3	Corynebacterium phage C3PO	171
Ribonucleotide reductase	SEA_DARWIN_74	Corynebacterium phage Darwin	648
Ribonucleotide reductase large subunit	phiAbaA1_082	Acinetobacter phage vB_AbaM_phiAbaA1	968
Ribonucleotide reductase large subunit (EC 1.17.4.1)	SEP1_061	Staphylococcus phage phiIBB-SEP1	705
RIIA-like protein	153 SEA_ANNADREAMY_153	Streptomyces phage Annadreamy	639
RIIB-like protein	164 SEA_COMRADE_164	Streptomyces phage Comrade	336
RIIB-like protein	SEA_MILDRED21_228	Streptomyces phage Mildred21	326
RNA-binding protein		Streptomyces phage BRock	523
Scaffold protein	PHL179M00_05	Propionibacterium phage PHL179M00	184
Scaffolding protein	SEA_LILBANDIT_5	Propionibacterium phage LilBandit	184
Scaffolding protein	MOYASHI_5	Propionibacterium phage Moyashi	184
Secreted transglycosylase	Quidividi_034	Staphylococcus phage Quidividi	220
SF4 helicase domain-containing protein		Propionibacterium phage pa28	287
Structural protein	Pepy6gene012	Rhodococcus phage ReqiPepy6	115
Structural protein	AB9_053	Acinetobacter phage vB_AbaM_B9	178
Tail assembly chaperone	SEA_C3PO_27	Corynebacterium phage C3PO	273
Tail assembly chaperone	AB9_056	Acinetobacter phage vB_AbaM_B9	131
Tail assembly chaperone	SEA_LEVIOSA_13	Propionibacterium phage Leviosa	227
Tail length tape-measure protein		Propionibacterium phage pa33	921
Tail length tape-measure protein		Propionibacterium phage pa63	921
Tail lysin	SEP1_028	Staphylococcus phage phiIBB-SEP1	1401
Tail lysozyme	30 TAEYOUNG_30	Arthrobacter phage TaeYoung	110
Tail protein	32 BARRETLEMON_32	Arthrobacter phage BarretLemon	427
Tail protein	19 JAWNSKI_19	Arthrobacter phage Jawnski	448
Tail protein		Moraxella phage Mcat20	1460
Tail protein		Staphylococcus phage phiIPLA-C1C	1151
Tail protein	27 PRINCESSTRINA_27	Arthrobacter phage PrincessTrina	645
Tail protein	35 KELLEZIO_35	Arthrobacter phage KellEzio	1704
Tail protein	vB_RpoS-V16_51	Ruegeria phage vB_RpoS-V16	1614
Tail protein		Actinomyces virus Av1	731
Tail sheath	14 JAWNSKI_14	Arthrobacter phage Jawnski	482
Tail sheath	15 MARTHA_15	Arthrobacter phage Martha	482
Tail sheath protein	AB9_051	Acinetobacter phage vB_AbaM_B9	381
Tail sheath protein	SEA_CHOCOLAT_18	Arthrobacter phage Chocolat	482
Tail spike protein	CPT_Mater149	Bacillus phage Mater	663
Tail spike protein	Eldridge_087	Bacillus phage Eldridge	663
Tail-like protein	Shpa_19	Paracoccus phage Shpa	1072
Tape measure protein	PHL141N00_14	Propionibacterium phage PHL141N00	921
Tape measure protein	PHL067M09_14	Propionibacterium phage PHL067M09	921
Tape measure protein	SEA_LUCY_14	Arthrobacter phage Lucy	853
Tape measure protein	PROCRASS1_14	Propionibacterium phage Procrass1	921
Tape measure protein	NIKTSON_26	Arthrobacter phage Niktson	1529
Tape measure protein	AB9_058	Acinetobacter phage vB_AbaM_B9	681
Tape measure protein	22 SEA_CHEESY_22	Arthrobacter phage Cheesy	1492
Tape measure protein	Gsput1_18	Gordonia phage Gsput1	1431
Tape measure protein	PHL082M02_14	Propionibacterium phage PHL082M02	921
Tape measure protein	KEIKI_14	Propionibacterium phage Keiki	921
Tape measure protein	17 SEA_FROKOSTDAME_17	Gordonia phage Frokostdame	1824
Tapemeasure protein	SEA_AQUARIUS_14	Propionibacterium phage Aquarius	921
Terminase	PHL009M11_02	Propionibacterium phage PHL009M11	503
Terminase large subunit	6 SEA_HOTFRIES_6	Streptomyces phage HotFries	581
Terminase large subunit	SEA_TIMINATOR_2	Arthrobacter phage Timinator	489
Terminase large subunit	BiPBO1_02	Brucella phage BiPBO1	562
Terminase large subunit	KEIKI_2	Propionibacterium phage Keiki	503
Terminase large subunit	8 CIRCUM_8	Arthrobacter phage Circum	584
Terminase large subunit	MRAK_2	Propionibacterium phage MrAK	503
Terminase small subunit	BiPBO1_01	Brucella phage BiPBO1	133
Terminase small subunit	3 SEA_MEMENTOMORI_3	Microbacterium phage MementoMori	194
Terminase small subunit	SEA_C3PO_1	Corynebacterium phage C3PO	174
Thioredoxin	SEA_DARWIN_54	Corynebacterium phage Darwin	98
Thymidylate synthase	SEA_ZION_9	Corynebacterium phage Zion	257
Thymidylate synthase	SEA_LUCKYBARNES_41	Brevibacterium phage LuckyBarnes	517
Thymidylate synthase	CB7_206	Pectobacterium phage vB_PatM_CB7	226
Thymidylate synthase	109 PBI_COUNT_109	Microbacterium phage Count	232
Toprim domain-containing protein	30 P100D_30	Propionibacterium phage P100D	223
Transposase	SEP1_056	Staphylococcus phage phiIBB-SEP1	369
Tryptophan synthase beta superfamily protein	2 SEA_ALANGRANT_2	Mycobacterium phage AlanGrant	289

## Discussion

The skin is usually considered a hostile environment for nucleic acids, particularly RNA, because of the presence of nucleases, but inside EVs or other types of complexes, nucleic acids are likely to be protected. EVs have now been found in most biofluids, including sweat [16, 17], but proper inventory of sweat nucleic acids is still needed to determine the potential usefulness of sweat for nucleic acid biomarker discovery.

The most covered DNA and the most represented mRNA in the sweat samples were from mitochondrial origin. Mitochondria have been shown to be released by cells during oxidative stress [21], and to be transported in EVs [22], but we could not detect any intact mitochondria by TEM in our preparations. Mitochondrial protein have been reported in melanoma EVs [23] so we can speculate that the mitochondrial DNA in our samples was a result of mitophagy, which is a normal part of the skin’s aging process [24, 25]; Alternatively, in context of the skin, mitochondria may also be transported out of melanocyte during melanosome release, as the two organelles are tightly bound during melanogenesis [26].

On the other hand, total nuclear DNA is more sparsely represented with very few counts from coding genes while some unannotated regions are highly over-represented in all four samples, indicating that these sequences may not be randomly secreted. DNA as EV cargo is still controversial [27], as in most cases it is not protected from DNAse and might be just sticking to the EV surface, although there are exceptions like giant oncosomes [28], or physiological process to protect cells from activation of DNA-damage-response and cell cycle arrest or apoptosis [29] and parasite like plasmodium use DNA-loaded EVs to prime host cells for infection [30].

It is unclear how the characterized sweat DNA is associated with sweat EVs, but it is very likely that some of it is associated with apoptotic bodies resulting from sebum secretion collected by the flow of sweat during exercise, which is consistent with the presence of nucleic acids from bacteria typical of the sebaceous glands, such as *Propionibacterium (Cutibacterium) acnes* and their associated bacteriophages.

Small RNA sequencing with small sample quantity is challenging, resulting in a large proportion of unmapped reads. EVs have been shown to have RNA both on their surfaces and inside, but most reports show that the larger RNA species (larger than 200 bp) are absent altogether. Best studied EV-associated RNAs are miRNAs. Even though we did not use RNAse, obtaining detectable amount of RNA proved challenging. Even with a small RNA protocol, our samples were mostly tRNAs and miscRNA with a small representation of miRNAs. We were nevertheless able to confirm the presence of even the lowest represented miRNA in most samples tested using qPCR. Based on our list of miRNA we were able to identify miR21-5p and miR26a-5p as regulated by exercise [17].

Using an unbiased sequencing approach with individual samples confirmed the predominance of tRNA, rRNA and miscRNA observed in many other EV RNA studies [31]. It was more surprising to identify more than 500 protein coding RNA detectable in at least 9 samples and to find that some of them are spanning several spliced introns. Previously published reports point to several explanations for the presence of mRNA. The mRNA exist in co-purified protein complexes [31] or bound to secreted ribosomes [32], which partly protect the mRNA from degradation. It was also interesting to see that a large proportion of the EV-enriched sweat mRNAs are common to the transcriptome of the human eccrine gland [33]. GO analysis mostly shows enrichment in ribosomal components and translation but no clear cellular origin as most of the mRNAs identified tend to be ubiquitously expressed. While it is possible that EV-enriched sweat indeed contain full length functional mRNAs it is more likely reflecting the functional status of the cells that release EVs to sweat, without any particular function of its own.

Of further interest is the presence of microbiome derived particles. The most abundant phyla identified by NGS analysis were Proteobacteria, Actinobacteria, Firmicutes and Bacteroidetes, which are usually found on human arms, hands, and axilla. Proteobacteria are dominant on face and torso [34]. City scale metagenomics studies like the one performed in New York city underground system revealed a lot of information about underground users and their skin microbiome and highlighted that each individual sheds genetic information from their skin, both from their own genome and from their own microbiome, which can be retrieved for analysis [35]. Our data is consistent with that large-scale study result, and the conclusion that the human DNA collected in these metagenomics studies is most likely derived from human sweat.

Further studies are needed to determine if any of the RNAs found in our study are of clinical value, SNPs in some mRNA identified are associated with known diseases and could be further studied. For example, CALM2, which mRNA was identified in all the samples, has SNPs associated with cardiac arrhythmia and sudden death of young people after exercise [36]. Larger scale studies could determine if it is possible to identify clinically associated variants from sweat RNA. Other abundant misc. RNAs such as RNY1,3 and 4 are also considered to have diagnostic potential for inflammatory diseases [37] and cancer [38].

## Study limitations

Sweat as a biofluid presents many challenges, and the most important ones in the frame of this study are that the human skin is exposed to the environment, and it is an ecosystem where many organisms live. Skin secretions, including sweat, are metabolised by skin microbes, and the skin microbes secrete their own products, including outer membrane vesicles. The non-human nucleic acids we identified originated primarily from the skin microbiota, but also possibly from clothing and working surfaces, or from the collection material. Distinguishing contaminant nucleic acids in human sweat is especially challenging, since contaminants introduced in the process of sample handling are also mainly derived from human skin secretions. RNA extraction columns have been shown to contain contaminant RNA, and a small RNA sequencing data set available from data repository also show the presence of these contaminating RNAs [39]. Capturing total EVs from biofluids is still not possible by standard methods, and the choice of approach taken here therefore represents a trade-off between quantity/diversity and purity. The ExoRNEasy kit captures EVs on a filter and then proceeds directly to on-filter lysis for RNA isolation. For a biofluid like sweat, in which the EV quantity depends on individual factors and also ambient temperature, hydration status and length and intensity of exercise, capturing particles appeared to be a good choice for comparing heavy and light sweat producers. But as humans are constantly secreting a small quantity of eccrine sweat, alternative methods of collection might be more appropriate for biomarker development, including for sport-associated studies.

In line with MISEV2018 recommendations [40] and because we are aware that our type of preparation is not of high purity, we have used the term sweat particles, or EV-enriched preparation in this report. We are describing preparation methods in detail in the method section and have submitted the study to EV-track (EV-TRACK ID: EV210083), EV-metric 14% for the DNA preparation and 50% for the RNA study.

## Conclusions

Our data shows that that sweat particles are a good source of nucleic acid as has been reported for other biofluids. As the skin surface offers a site for non-invasive and real-time sample collection our study opens the path for future sweat EV biomarkers discovery.

## Methods

### Volunteers

Adult volunteers were recruited among persons of different ethnic background residing in Northern Finland in Oulu area (**Table** **1**). Volunteers were given information about the study and provided limited health and fitness self-assessment in a form and informed consent. Ethical permission (EETTMK:110/2015) was granted by the ethical committee of Oulu University medical School according to the Finnish Medical Research Act (488/1999). Volunteers were asked to avoid using soap and perfume for 24 h before the exercise and to shower with water only for 15 min immediately before exercise to remove dust and other environmental contaminant residues from the skin. These studies were performed according to the Declaration of Helsinki on research involving humans. The study protocol named RUBY was approved by the Ethical Committee at the Northern Ostrobothnia Hospital District in Oulu under Study Diary Number 110/2015. Participants in the study were given information about the study and signed informed consent forms approved by the ethics committee.

### Pooled sweat processing and nucleic acid analysis

We first collected large amount of sweat from 13 people of both genders aged 26 to 56 years, during a 40 min biking exercise (Fig. 1). Collected sweat was kept at -20 degrees until processing. After thawing, pooled sweat was filtered on 0,45 μm Milipore PES filters, then centrifuged for 2 h at 108000 x g in an Avanti J-30I centrifuge (Beckman) using JA30–50 rotor. Pellets were resuspended in 1 ml PBS without CaCl_2_ and MgCl_2_ pH 7, and 200 μl were used for DNA extraction (corresponding to approximately 80 ml of sweat) with QIAamp blood DNA mini-kit by Qiagen [41], (Fig. 1 left) remaining sample was used for RNA extraction. Concentration was measured using Qubits DNA HS assay kit (ThermoFisher). For buffer exchange and concentration Zymo DNA & Clean-5 columns (Zymo Research) were used with modified protocol (5 volume of binding buffer and elution with 56 °C pre-heated H_2_O). Samples from 3 individual donors (Table 1 top panel) were prepared in similar way. One ng of DNA was used for PE library construction using Nextera XT library preparation kit (Illumina) according to manufacturer instructions. Libraries were run on NextSeq550 sequencer (Illumina) with 151 cycles in Biocenter Oulu sequencing core facility.

The remaining sample corresponding to 80% of the original sweat volume were used for RNA extraction with an ExoRNeasy kit (Qiagen) according to manufacturer’s instructions.

Total RNA concentration was measured with Qubits RNA HS and profiled on Bioanalyzer 6000 Pico chips (Agilent). Pooled EV-enriched sweat RNA-seq RNA library was made using Ion Total RNA-Seq Kit v2 (Thermo Scientific) following instructions for small RNA libraries. In this case, purification beads were included in kit and used to remove adapter dimers. Final libraries were checked on a Bioanalyzer with High Sensitivity DNA kit (Agilent). Sequencing of libraries was done with Ion PGM Hi-Q OT2 Template (200 bp protocol), Ion PGM Hi-Q Sequencing Kit and Ion PGM 318 chip kits (Thermo Scientific).

### Individual sweat collection and processing for nucleic acid processing and analysis

Sweat was collected from the upper body, arms and torso using plastic raincoat (Transpen Oy, Kerava, FI) and disposable gloves VETbasic (15,364, Kerbl, Buchbach, Germany). If volunteers sweated heavily from their head, dripping sweat was collected in the head cover of the coat and pooled with the rest. Volunteers used exercise bike (ProSpinner spinning bike, Karhu) indoors for 30 min (Fig. 1 right). After exercise sweat was collected by cutting tip of gloves and cutting insert in ventral area to pipet fluid with sterile disposable pipet. Sweat was passed through 40 μm strainer, then through 0,8 μm filter (Millipore). If not immediately processed for nucleic acid extraction, filtered sweat was stored at -20oC in sterile Falcon tubes.

Filtered sweat was concentrated on Centricon Plus-70 centrifugal filter (100 k cut-off), according to instructions by manufacturer. Concentrated sweat RNA was extracted using exoRNeasy kit (Qiagen).

RNA-seq libraries were made using NEBNext Small RNA kit (New England Biolabs). After 16 cycles of PCR amplification, Libraries were checked with Bioanalyzer using DNA 1000 chips (Agilent). Before size selection on pippin blue (Thermo Fisher) libraries were mixed in 2 pools according to DNA yield. Size selection was set to collect fragment from 145 bp–200 bp. Size selected Pools were amplified an additional 5 cycles, purified wit PCR clean-up kit (Qiagen) and quantified by KAPA PCR kit (Roche). After dilution adjusting for library number in each pool, they were loaded on NextSeq550 (Illumina) and run 51 cycles.

### Bioinformatics analysis

#### DNA

DNA fastQ files were checked with FastQC [42], merged using PEAR [43], merged and unmerged reads were aligned with BWA [44] against human genome HG38. Ensembl 94 annotation was used to intersect reads with functional elements. Coverage percentages for each chromosome was calculated as length of mapped reads per chromosome divided by length of chromosome.

#### RNA

Reads from different lanes were first merged into single fastq files and a QC was performed [42]. Then, low quality bases and adapter sequences were trimmed with trimmomatic [45] followed by another QC with FastQC. Trimmed reads were then mapped with Bowtie [46] against GtRNAdb high confidence tRNA sequences [47] and reads that map against tRNA sequences were also filtered out. The remaining reads were then mapped again with Bowtie against the human genome HG38, and further processed wit Cufflinks [48] and Cuffmerge to prepare a joint annotation file that contains then both known and novel genes. This annotation file as well as an annotation file for miRNAs from miRBase [49, 50] and Human piRNA sequence v2.0 from piRBase [51] was then used to quantify the expression with cufflinks and featureCounts [52]. For quantification, only exonic counts were taken into account. Alignment with STAR (Spliced Transcripts Alignment to a Reference ([53]) was done using Chipster at https://chipster.csc.fi/ [54].

Reads that could not be mapped against the HG38 genome were then de-novo assembled to contig level using MEGAHIT [55]. These contigs were then blasted against the NR database with DIAMOND [56], and for the identified proteins the corresponding IDs were extracted. Further, with Kraken [57] a taxonomic identification for the unmapped reads was performed and the results were visualized using Krona [58].

### GO analysis

List of genes with FPKM values 25 or over were put into geneontology.org for enrichment analysis ( [59], the gene ontology consortium 2019) using Fisher’s exact test with Bonferroni correction for multiple testing and GO annotation with enrichment value 4 or over were visualized using REVIGO [60], dispensable GO terms were omitted.

### miRNA QPCR

1,5 ng of RNA was used for cDNA synthesis using miRCURY LNA RT-PCR kit (Qiagen).

Following LNA primers were used for QPCR using SYBR Green III master mix (Agilent) miRCURY LNA miRNA QPCR Assay: miR24-3p (YP00204260), miR99a-5p (YP00204521), miR193 (YP00204226), miR-21-5p (MS00009079), miR-26a-5p (MS00029239), miR320b (MIMAT0005792), U6 snRNA (X59362).

### RT-PCR

cDNA was made from 5 ng of RNA using VILO or Maxima H- first strand cDNA synthesis kit with DS DNAse (Thermo Fisher). After 1/2 dilution cDNA was amplified using AmpliTaq Gold and specific primers:

**Table:** 

Gene	forward primer	reverse primer
14–3-3 Protein Epsilon (YWHAE)	ACAGAACTTCCACCAACGCA	ATTCTGCTCTTCACCGTCACC
Ferritin Light Chain (FTL)	GGACCCCCATCTCTGTGACT	AGTCGTGCTTGAGAGTGAGC

PCR conditions: 95oC 5 min, 60oC 20s,72oC 20s, 95oC20s, 40 cycles. Products were analyzed on 2% agarose gel, stained with midori green and photographed. PCR products were purified using Qiagen minElute columns (Qiagen) and sequenced in Biocenter Oulu sequencing core facility using capillary sequencing with BigDyeTERminator v1.1 cycle sequencing (ABI) and ABI3500xL Genetic Analyzer.

### Electron microscopy

The immunoelectron microscopy was performed using biotinylated anti-CD9 antibody as a primary antibody at a 1:10 dilution. Vesicles were deposited on a Formvar carbonated grid (glow-discharged). The grids were incubated in blocking serum (1% BSA (bovine serum albumin) in PBS). Afterwards, the grids were incubated for 20 mins with the primary anti-CD9 antibody (Miltenyi Biotec), followed by the secondary anti-biotin antibody for 20 min and finally the protein A-gold complex (PAG 10 nm) for 20 min. Samples after immunonegative staining as well as after negative staining with 2% uranyl acetate were examined using a Tecnai G2 Spirit transmission electron microscope (FEI, Eindhoven, The Netherlands) and images were captured with a charge-coupled device camera (Quemesa, Olympus Soft Imaging Solutions GMBH, Münster, Germany). Anti-CD63 antibody for immuno-TEM was used at dilution 1:50 (Abcam ab193349) and polyclonal anti-Glypican 1 antibody (PA5–28055, ThermoFisher) at dilution 1:100.

For preparing plastic sections concentrated sweat was filtered on 0.45 um Minisart filter (Sartorius), then sweat EVs were stained with CellVue Claret Far Fluorescent Cell Linker Midi Kit (MIDCLARET-1KT) according to manufacturer’s instructions. After staining, samples were centrifuged for 4–6 h at 120 K rpm k-factor = 16 at 4 °C Beckman TLA 120.2). Supernatants were removed, pellets fixed and plastic embedded in Biocenter Oulu electron microscopy core facility. Thin sections were observed with Tecnai G2 Spirit electron microscope.

### Western blotting

EV samples were diluted in 5 X Laemmli loading buffer and proteins were separated on 10% SDS PAGE gel, then transferred to nitrocellulose membrane. Anti-CD63 (Abcam Ab193349; 1:500 and Santa Cruz H-193, sc-15,362; 1:1000 dilutions), GM130 (Cell Signaling Technology, #12480; 1:1000) and Ago2 (Abcam ab32381; 1:1000) antibodies were used for detection.

### Nanoparticle tracking analysis

Nanoparticle tracking analysis (NTA) was performed using a NanoSight NS300 (NanoSight Ltd., Amesbury, UK) equipped with a 405 nm laser. At least three 40 s videos were recorded of each sample with camera level and detection threshold set up at 13. Temperature was monitored throughout the measurements. Videos recorded for each sample were analyzed with NTA software version 3.1. (build 3.1.46) to determine the concentration and size of measured particles with corresponding standard error. For analysis, auto settings were used for blur, minimum track length and minimum expected particle size. Double distilled H_2_O was used to dilute the starting material.

## Supplementary Information

**Additional file 1: Supplementary Figure 1.** Bioanalyzer profile of RNA from individual samples of EV-enriched sweat. RNA analysis profiles for all subjects 1 ul of RNA was run on Agilent pico600 chips. **Supplementary Figure 2.** piRNA in individual samples. piRNA percentages in 20 individual samples, below table with normalized value for each sample. **Supplementary Figure 3.** TEM images, negative staining of EV-enriched sweat. Negative control image (PBS wash of collection glove processed as sweat samples), images of ExoEasy processed sweat from 4 different volunteers. **Supplementary Figure 4.** Nanoparticle Tracking analysis from Exoeasy prepared sweat, summary of 5 different isolations. **Supplementary Figure 5.** Western blots with protein from negative control (collection glove washed in PBS and processed with exoEasy as sweat), unbound material from ExoEasy column (flowthrough), ExoEasy eluted fraction (EV-enriched), and concentrated sweat (cut-off 100 kDa), were stained with anti-CD63 antibody (EV marker) and antibodies against non-EV markers Ago2 and GM130. A: membrane B: the same membrane probed with anti-CD63 antibdy. Fluorescent images were inverted, contrast and brightness adjusted to make bands visible. C: the same membrane probed with anti-Ago2 antibody. D: membrane E: the same membrane probed with anti-GM130 antibody. **Supplementary Figure 6.** Western blot, whole membrane from Fig. 4E. EV-enriched (ExoEasy isolation) sweat samples from three individuals were loaded (marked 2, 3, and 32). Region cropped is marked by the black frame. Original fluorescence image was inverted, then brightness and contrast were increased to make bands more visible. **Supplementary Figure 7.** Whole 2% agarose gel images for Fig. 10. Individual samples’ RNA was reverse transcribed and amplified with primers designed to amplify mRNA across exon-exon junctions. FTL band was cropped form each individual gel, cropped image is marked in blue box, YWHAE band was cropped from individual gels as indicated by red boxes.

**Additional file 2: Supplementary Table 1.** Transcripts identified in at least 18 samples.

**Additional file 3: Supplementary Table 2.** Comparison of top 1% RNA identified in Illumina and Ion Torrent.

## Data Availability

The RNA and DNA data described in this paper will be available from the European Nucleotide Archive (EMBL-EBI) under accession number PRJEB40112 https://www.ebi.ac.uk/ena/browser/home.

## Abbreviations

EV: Extracellular vesicle
NGS: Next-generation sequencing
TEM: Transmission electron microscopy
FPKM: Fragments per kilobase per million reads mapped
STAR: Spliced Transcripts Alignment to a Reference
RT-PCR: Reverse transcription polymerase reaction
qPCR: quantitative polymerase chain reaction
