# Supplementary material for: Characterization of nucleic acids from extracellular vesicle-enriched human sweat
Source: BMC Genomics. 2021 Jun 9;22:425. doi: 10.1186/s12864-021-07733-9 (PMC8188706; doi:10.1186/s12864-021-07733-9)
Supplement: Supplementary file 1 — Additional file 1: Supplementary Figure 1. Bioanalyzer profile of RNA from individual samples of EV-enriched sweat. RNA analysis profiles for all subjects 1 ul of RNA was run on Agilent pico600 chips. Supplementary Figure 2. piRNA in individual samples. piRNA percentages in 20 individual samples, below table with normalized value for each sample. Supplementary Figure 3. TEM images, negative staining of EV-enriched sweat. Negative control image (PBS wash of collection glove processed as sweat samples), images of ExoEasy processed sweat from 4 different volunteers. Supplementary Figure 4. Nanoparticle Tracking analysis from Exoeasy prepared sweat, summary of 5 different isolations. Supplementary Figure 5. Western blots with protein from negative control (collection glove washed in PBS and processed with exoEasy as sweat), unbound material from ExoEasy column (flowthrough), ExoEasy eluted fraction (EV-enriched), and concentrated sweat (cut-off 100 kDa), were stained with anti-CD63 antibody (EV marker) and antibodies against non-EV markers Ago2 and GM130. A: membrane B: the same membrane probed with anti-CD63 antibdy. Fluorescent images were inverted, contrast and brightness adjusted to make bands visible. C: the same membrane probed with anti-Ago2 antibody. D: membrane E: the same membrane probed with anti-GM130 antibody. Supplementary Figure 6. Western blot, whole membrane from Fig. 4E. EV-enriched (ExoEasy isolation) sweat samples from three individuals were loaded (marked 2, 3, and 32). Region cropped is marked by the black frame. Original fluorescence image was inverted, then brightness and contrast were increased to make bands more visible. Supplementary Figure 7. Whole 2% agarose gel images for Fig. 10. Individual samples’ RNA was reverse transcribed and amplified with primers designed to amplify mRNA across exon-exon junctions. FTL band was cropped form each individual gel, cropped image is marked in blue box, YWHAE band was cropped from individual gels as indic [file 12864_2021_7733_MOESM1_ESM.pdf]

Characterization of nucleic acids from extracellular vesicle-enriched human sweat

Genevieve Bart[1], Daniel Fischer[2], Anatoliy Samoylenko[1], Artem Zhyvolozhnyi[1], Pavlo Stehantsev[1], Ilkka Miinalainen[1], Mika Kaakinen[1], Tuomas Nurmi[1], Prateek Singh[1,4], Susanna Kosamo[1], Lauri Rannaste[3], Sirja Viitala[2], Jussi Hiltunen[3], Seppo Vainio[1]*

Affiliations

[1] Faculty of Biochemistry and Molecular Medicine, Disease Networks Research Unit, Laboratory of Developmental Biology, Kvantum Institute, Infotech Oulu, University of Oulu, 90014 University of Oulu, Finland
[2] Production Systems, Natural Resources Institute Finland (LUKE), 31600 Jokioinen, Finland
[3] Biosensors, VTT, Technical Research Center of Finland Ltd, Kaitoväylä 1, 90570 Oulu, Finland
[4] Current Address: Finnadvance, Aapistie 5, 90220 Oulu, Finland.

*Corresponding author: Seppo Vainio, seppo.vainio@oulu.fi

Supplementary figure 1

Bioanalyzer profile of RNA from individual samples of EV-enriched sweat. RNA analysis profiles for all subjects 1 ul of RNA was run on Agilent pico600 chips.

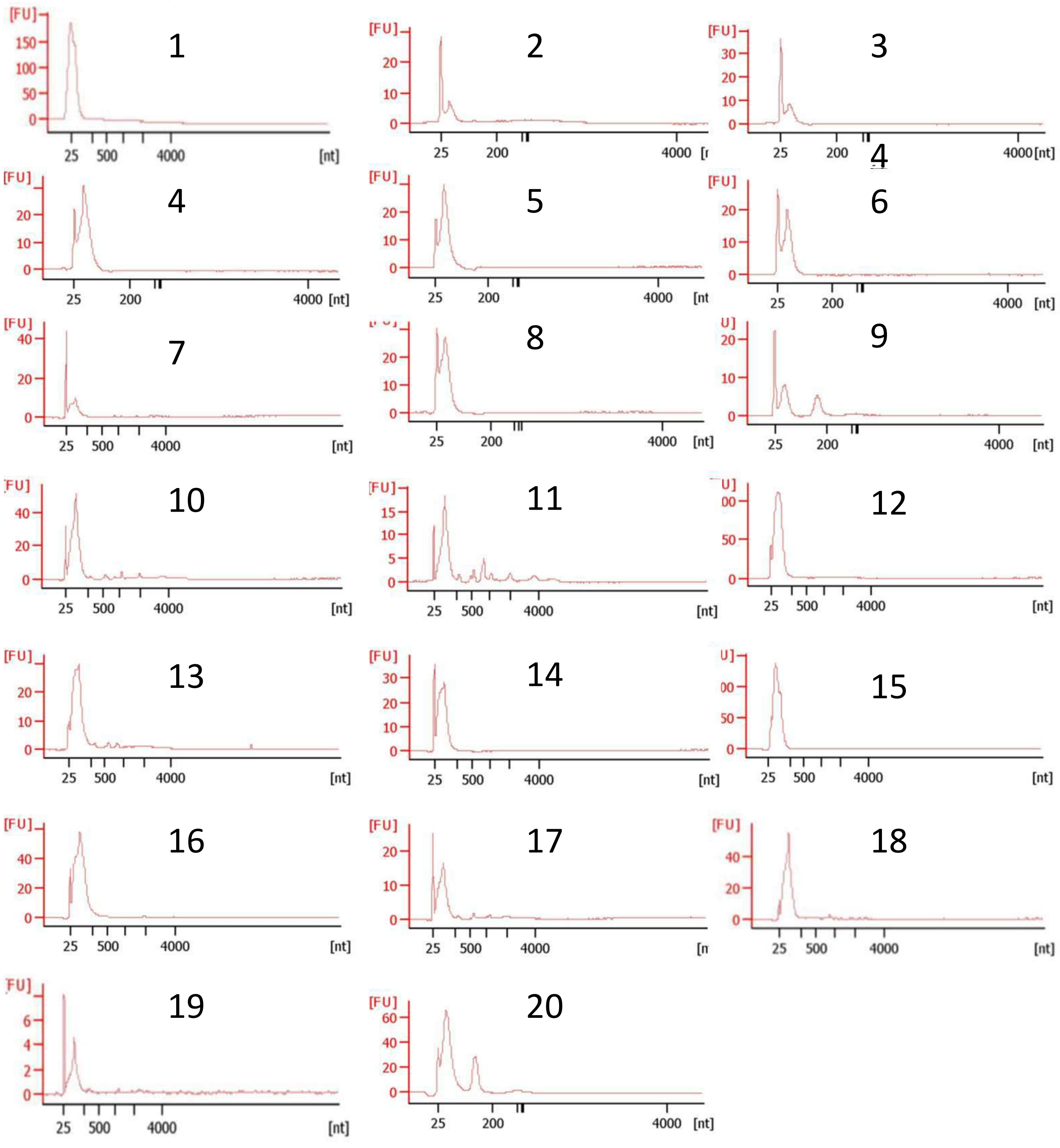

Supplementary Figure 2

piRNA in individual samples. piRNA percentages in 20 individual samples, below table with normalized value for each sample.

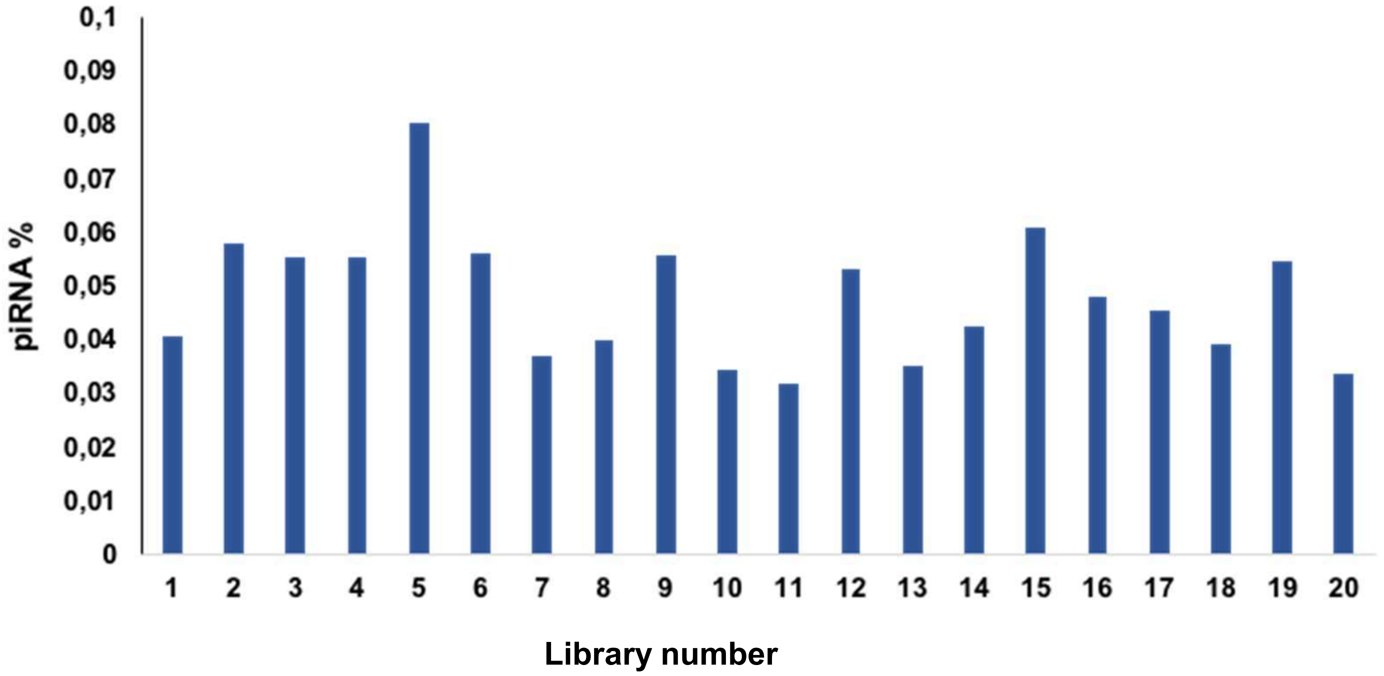

Geneid	1	2	3	4	5	6	7	8	9	10	11	12	13	14	15	16	17	18	19	20
piR-hsa-1898901	47,4	9,8	5,9	8,6	11,3	11,9	33,4	25,6	10,3	22,9	46,7	4,4	30,5	47,7	8,4	9,4	42,3	50,0	5,2	38,5
piR-hsa-2512273	33,2	9,8	8,2	9,6	7,9	7,6	33,4	35,8	9,2	22,9	22,0	9,8	37,6	23,9	5,1	4,1	29,1	53,8	9,3	34,4
piR-hsa-2845376	19,0	18,3	17,6	9,6	12,5	15,2	9,5	30,7	21,5	9,2	27,5	21,3	16,4	0,0	13,5	9,4	13,2	7,7	14,5	18,2
piR-hsa-3525320	35,6	43,9	47,0	49,8	73,7	42,2	45,4	17,9	66,6	27,5	16,5	40,0	35,2	35,8	48,9	40,7	39,7	46,1	48,8	36,4
piR-hsa-4371383	23,7	6,1	5,9	9,6	12,5	10,8	31,0	38,4	12,3	59,6	41,2	11,6	35,2	41,8	13,5	5,3	29,1	34,6	8,3	28,3
piR-hsa-4439011	23,7	8,5	24,7	9,6	19,3	23,8	33,4	28,1	24,6	18,3	22,0	19,6	35,2	41,8	13,5	25,3	60,9	53,8	17,7	22,3

piRNA in individual sweat samples:
Top: percentage of reads per library
Table piRNA from
http://www.regulatoryrna.org/database/piRNA/download.html
piRNA expressed with standardized value 5 (read in region/total amount of reads * 100000)

Supplementary Figure 3
TEM images, negative staining of EV-enriched sweat. Negative control image (PBS wash of collection glove processed as sweat samples), images of ExoEasy processed sweat from 4 different volunteers

Negative control :

collection glove was washed with 50 ml of 0,22µm filtered cell culture grade PBS, filtered with 0,8 µm milipore filter and concentrated on centricon 70 100 kDa colum, then prepared with exoEasy kit. Negative staining TEM image

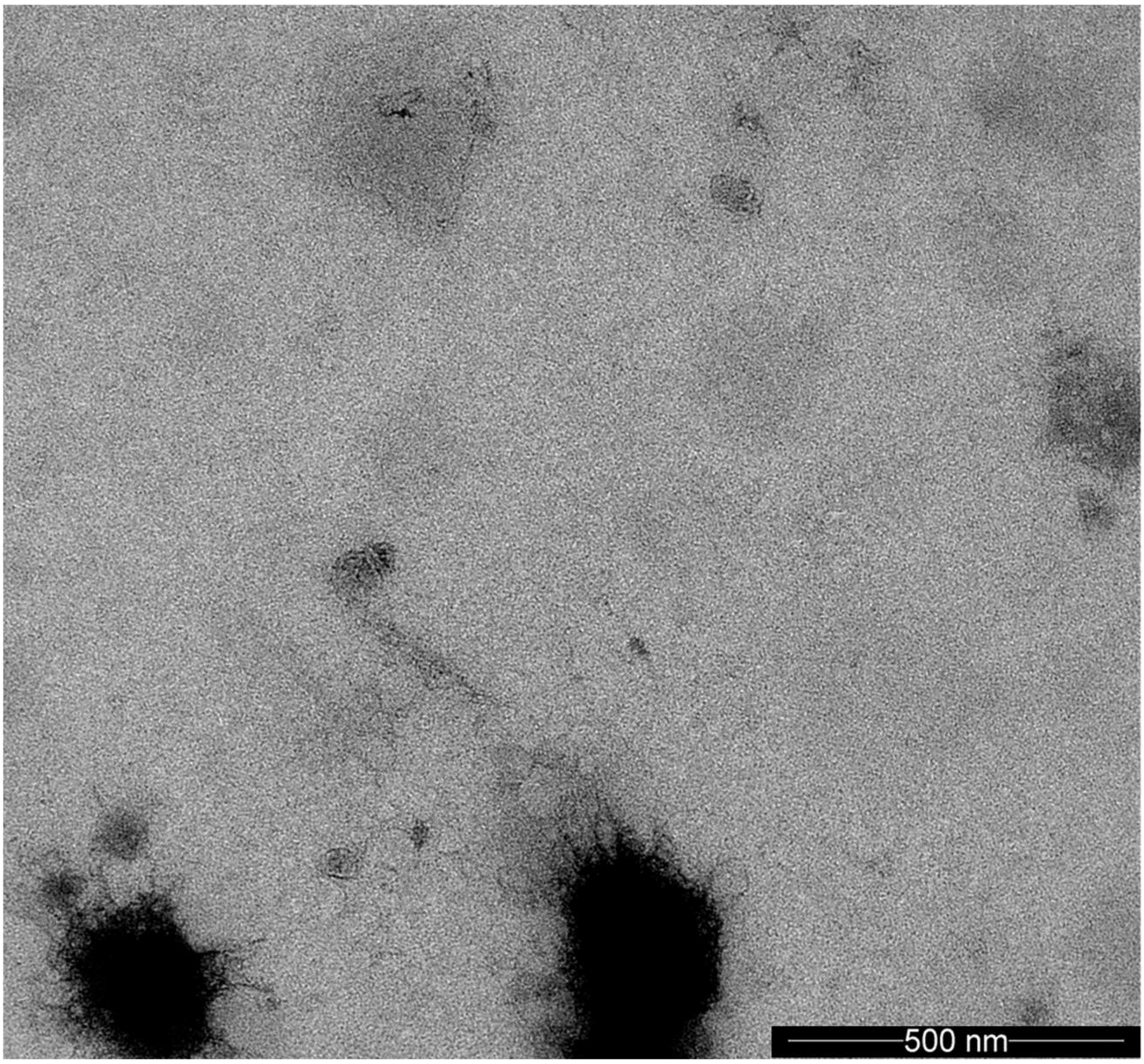

Supplementary Figure 3 continued

TEM images: negative staining of sweat particles

Sample number corresponding to table 1

Sample 2

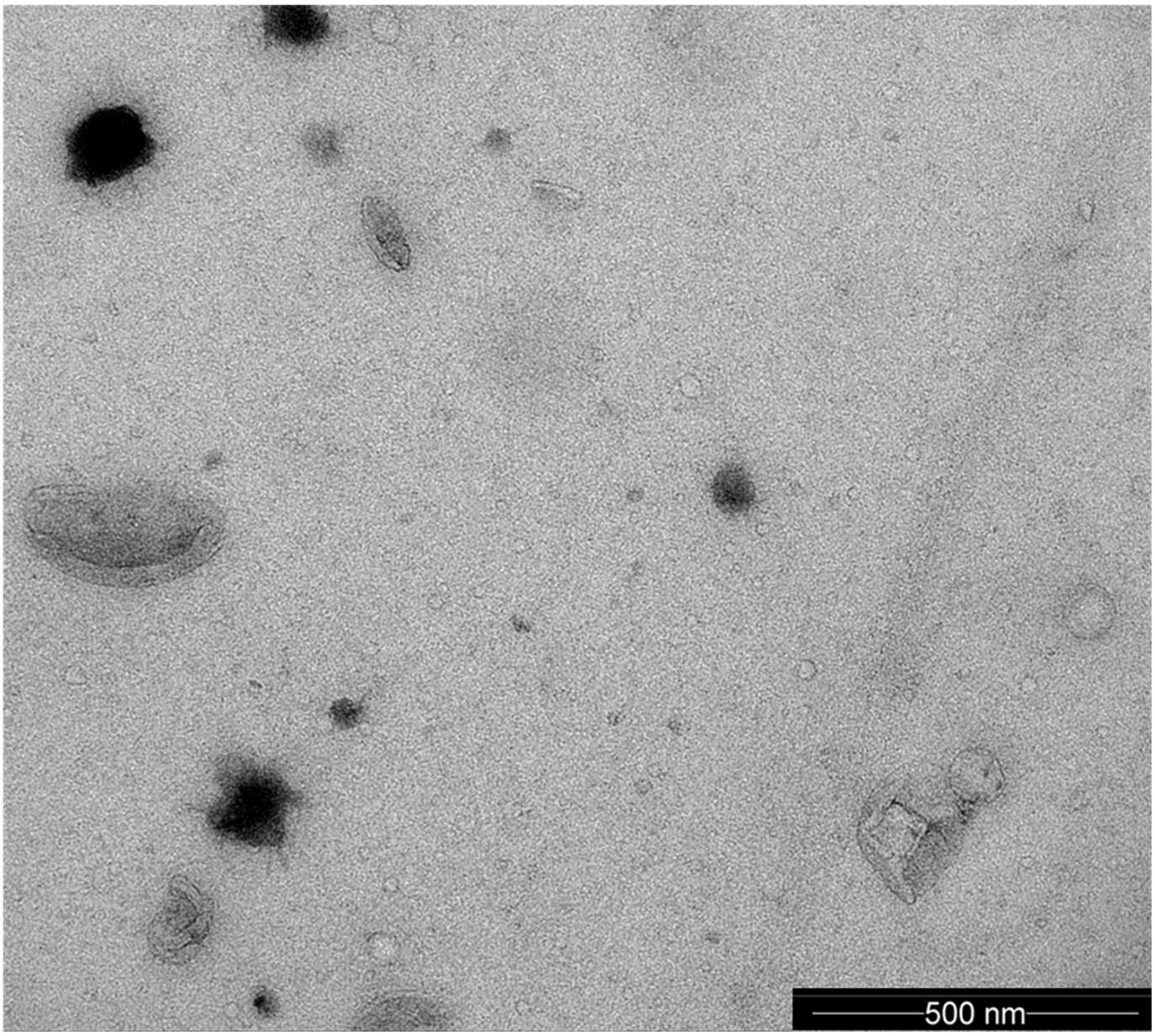

Supplementary Figure 3: Continued

Sample 6

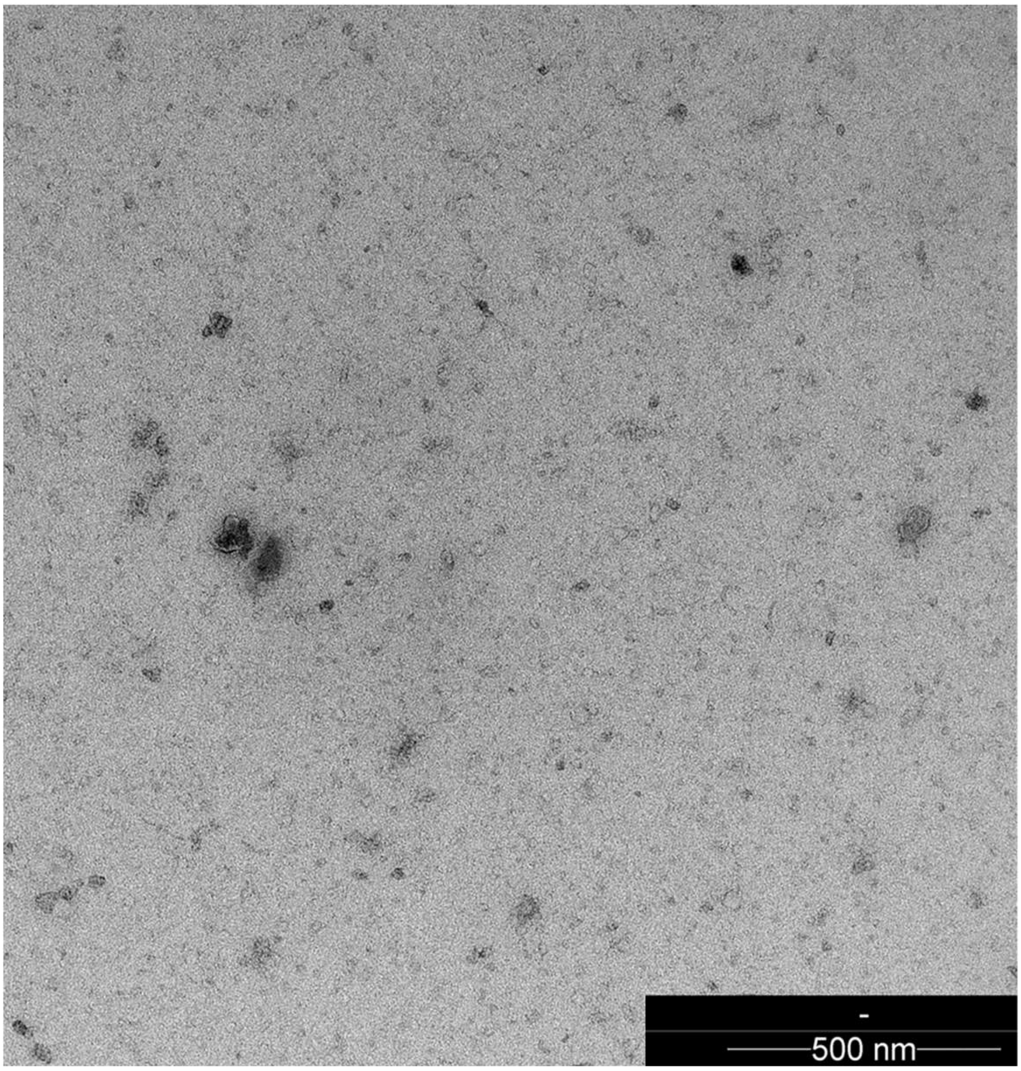

Sample 11

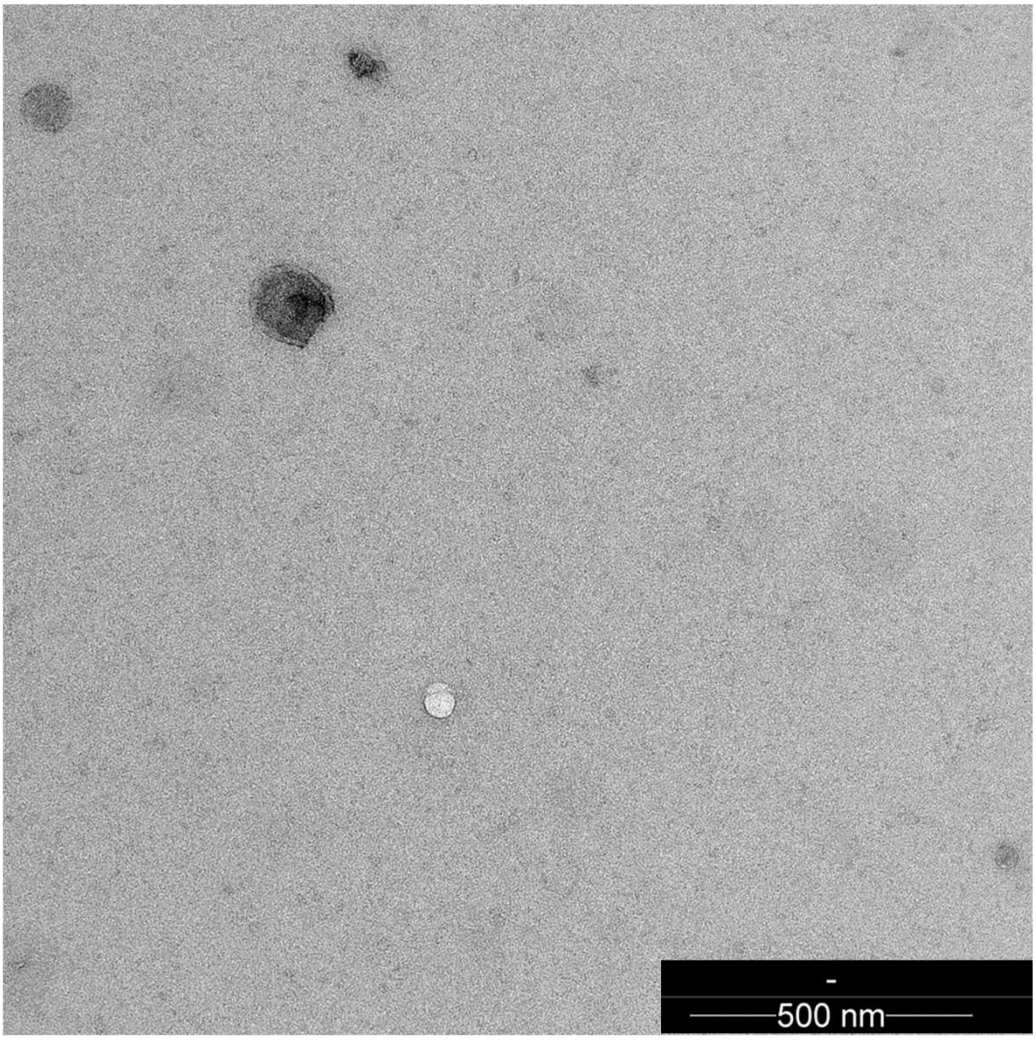

Supplementary Figure 3 continued

Sample 20

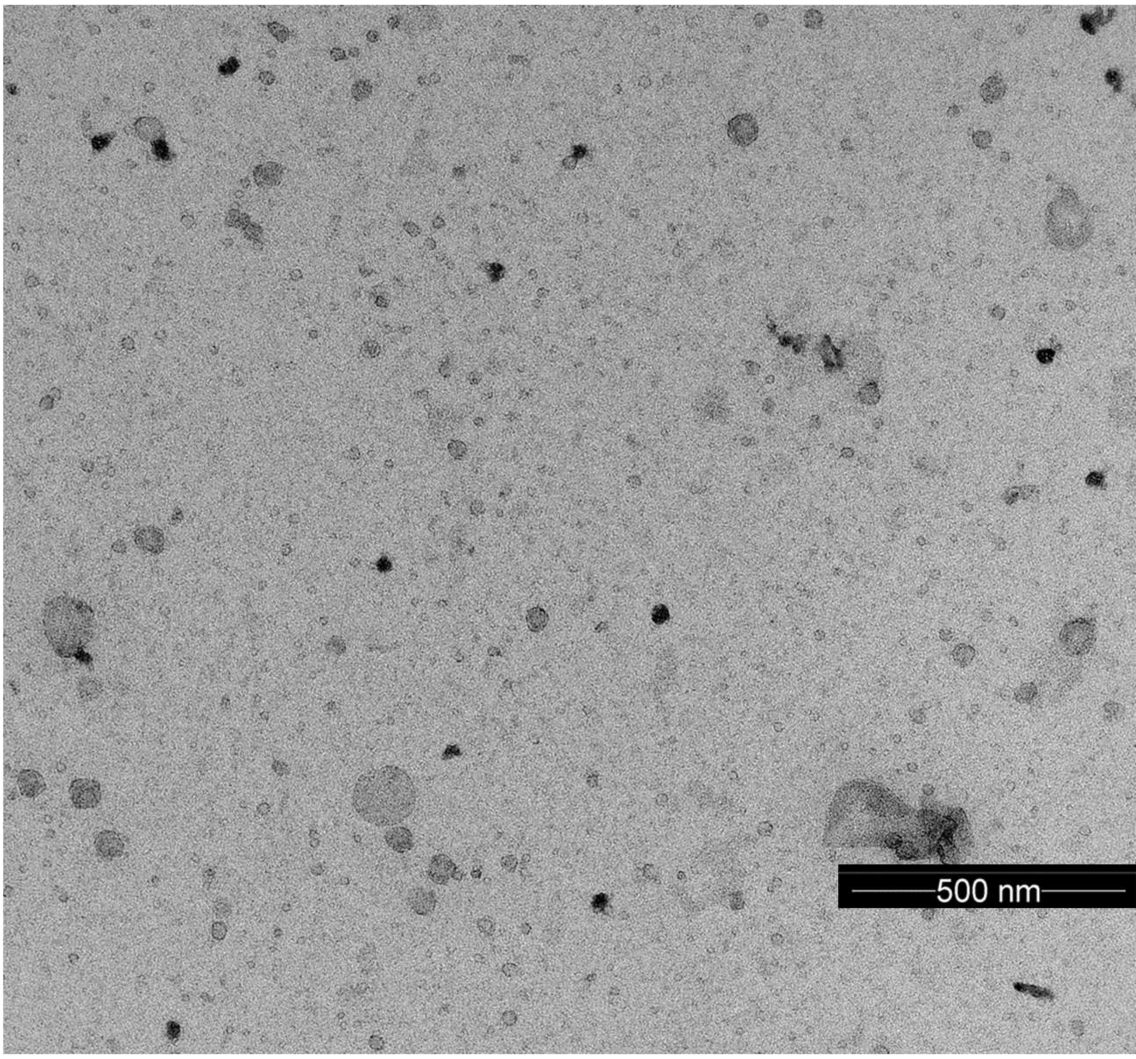

Sample 21

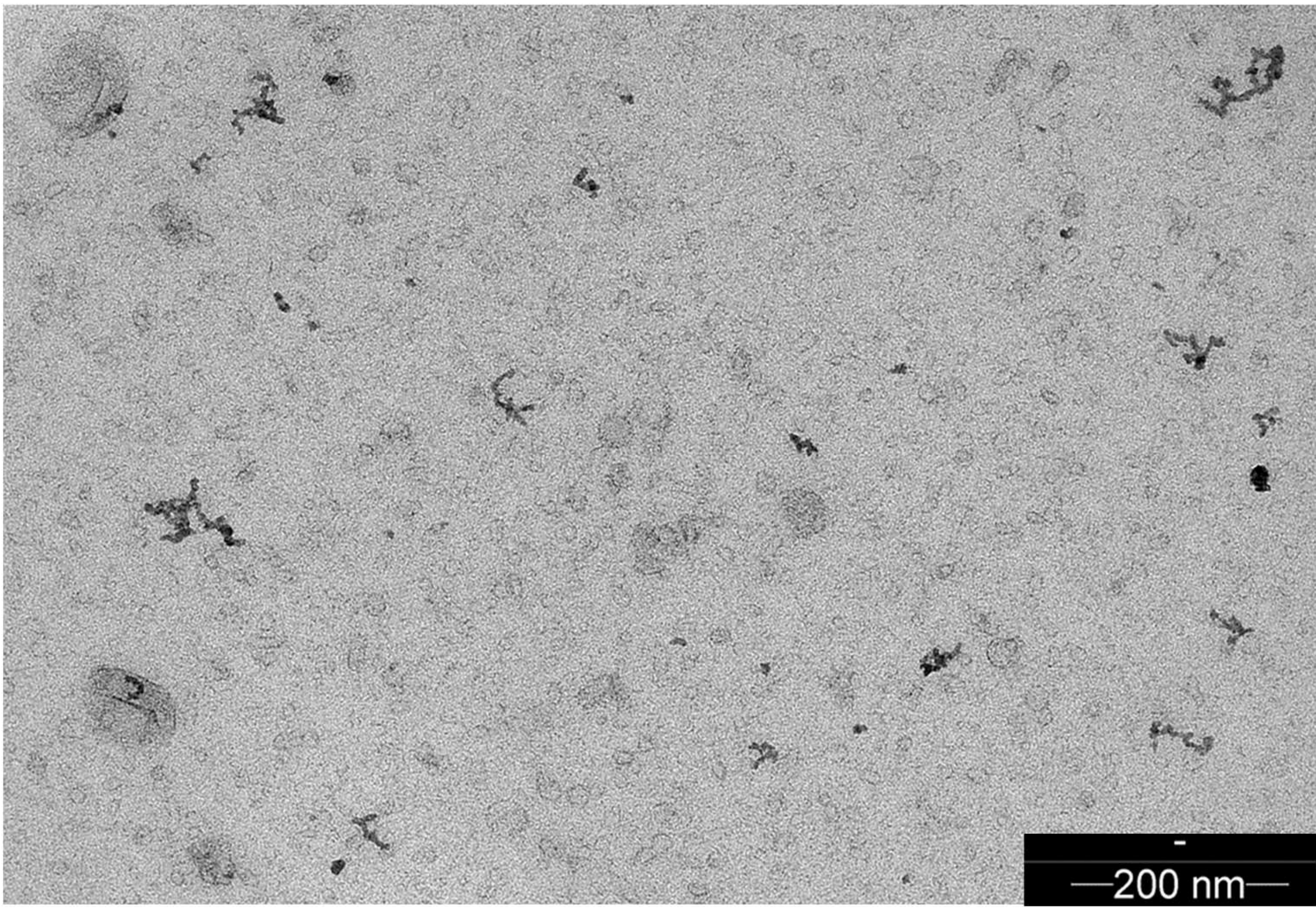

Supplementary Figure 4

Nanoparticle Tracking analysis from Exoeasy prepared sweat, summary of 5 different isolations.

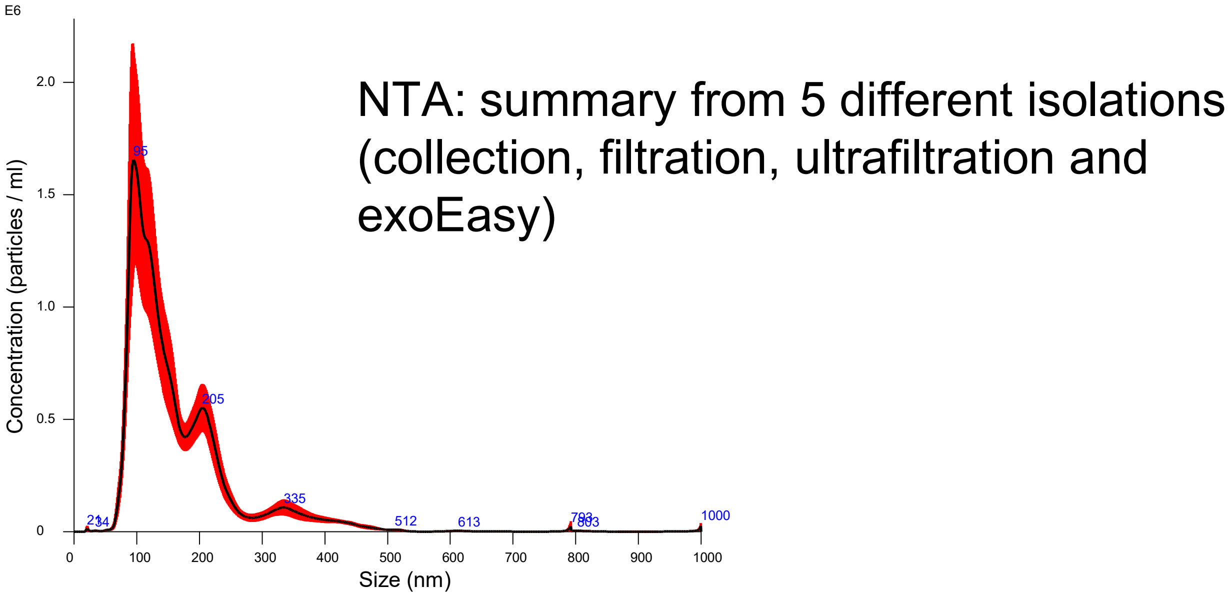

Supplementary Figure 5

Western blots with protein from negative control (collection glove washed in PBS and processed with exoEasy as sweat), unbound material from ExoEasy column (flowthrough), ExoEasy eluted fraction (EV-enriched), and concentrated sweat (cut-off 100kDa), were stained with anti-CD63 antibody (EV marker) and antibodies against non-EV markers Ago2 and GM130. A: membrane B: the same membrane probed with anti-CD63 antibdy. Fluorescent images were inverted, contrast and brightness adjusted to make bands visible. C: the same membrane probed with anti-Ago2 antibody. D: membrane E: the same membrane probed with anti-GM130 antibody.

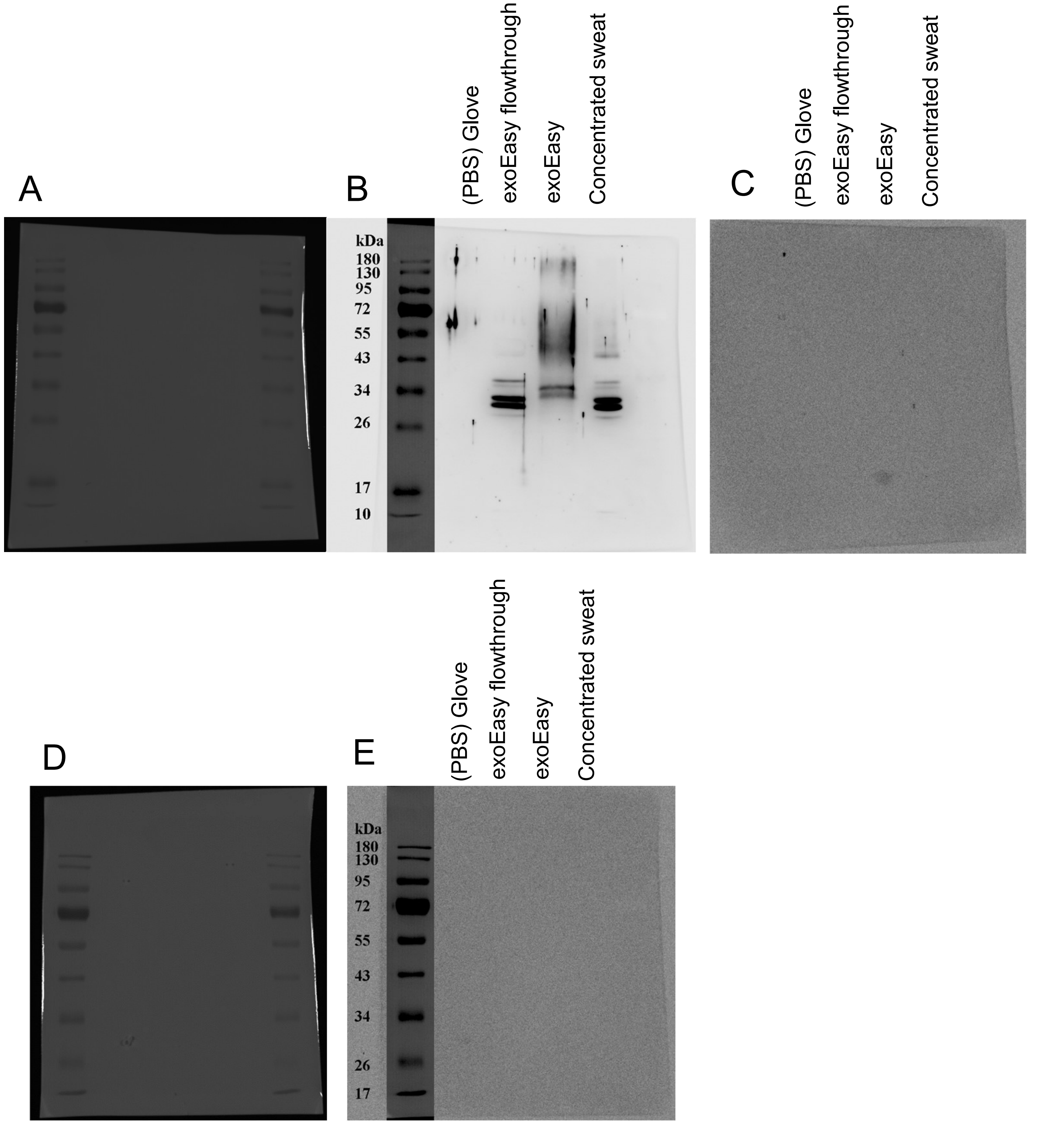

Supplementary Figure 6

Western blot, whole membrane from Figure 4E. EV-enriched (ExoEasy isolation) sweat samples from three individuals were loaded (marked 2, 3, and 32). Region cropped is marked by the black frame. Original fluorescence image was inverted, then brightness and contrast were increased to make bands more visible.

Samples 2 3 32

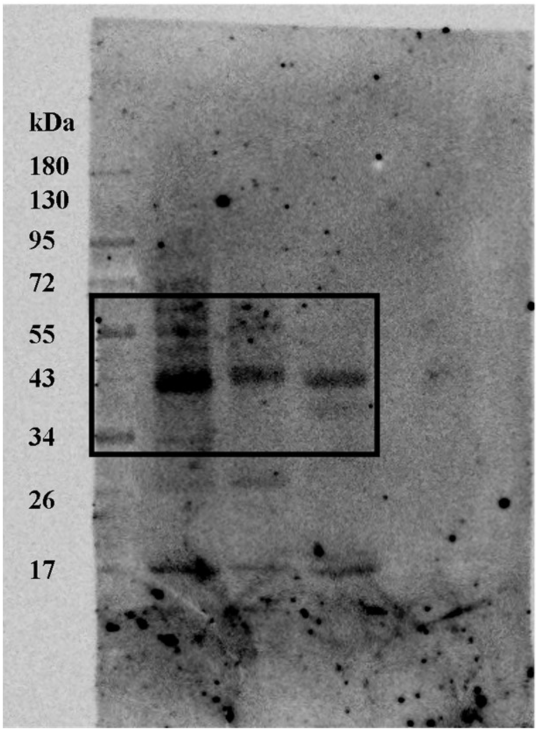

Western blot CD63 (Figure 4E)

Abcam, ab193349
original image was inverted and brightness and contrast were increased

Supplementary Figure 7

<u>Whole 2% agarose gel images for figure 10</u>. Individual samples' RNA was reverse transcribed and amplified with primers designed to amplify mRNA across exon-exon junctions. FTL band was cropped form each individual gel, cropped image is marked in blue box, YWHAE band was cropped from individual gels as indicated by red boxes.

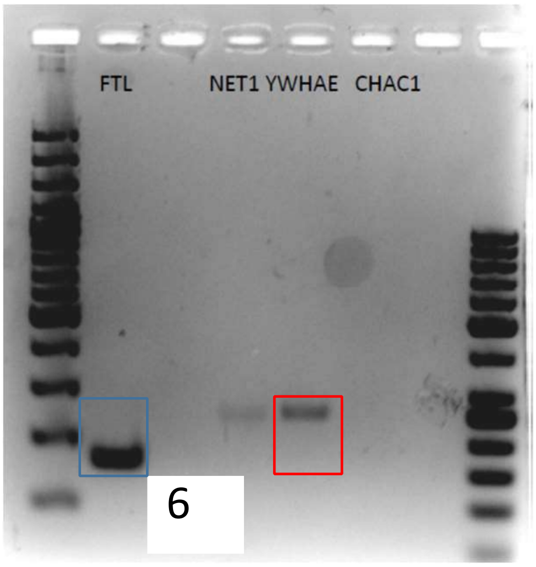

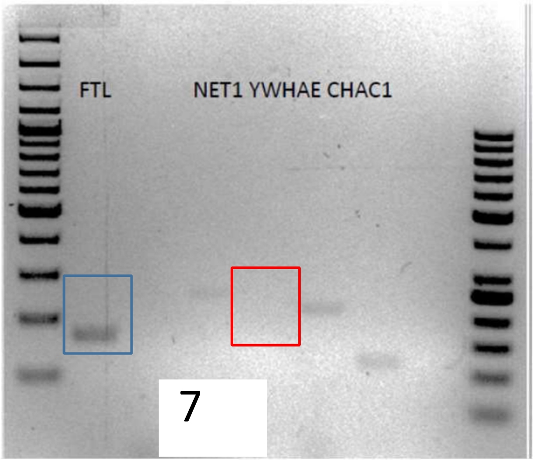

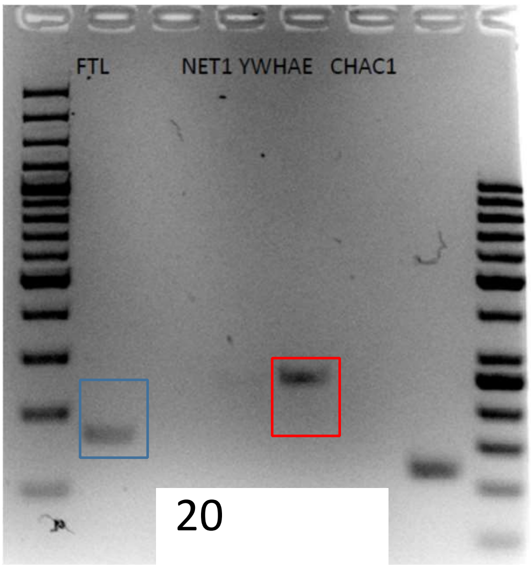

2% Agarose gel (Figure 10)

RT-PCR from 3 individuals

FTL Ferritin Light Chain

NET Neuroepithelial cell-transforming gene 1 protein

YWHAE 14-3-3 protein epsilon

CHAC1 Glutathione-specific gamma-glutamylcyclotransferase **1**

Last lane gel 20 :CSDE1: Cold shock domain-containing protein E1
